# Diverse origin of mitochondrial lineages in Iron Age Black Sea Scythians

**DOI:** 10.1038/srep43950

**Published:** 2017-03-07

**Authors:** Anna Juras, Maja Krzewińska, Alexey G. Nikitin, Edvard Ehler, Maciej Chyleński, Sylwia Łukasik, Marta Krenz-Niedbała, Vitaly Sinika, Janusz Piontek, Svetlana Ivanova, Miroslawa Dabert, Anders Götherström

**Affiliations:** 1 Department of Human Evolutionary Biology, Institute of Anthropology, Faculty of Biology, Adam Mickiewicz University in Poznan, Umultowska 89, 61-614 Poznan, Poland; 2 Department of Archaeology and Classical Studies, Stockholm University Wallenberglaboratoriet, SE-106 91 Stockholm, Sweden; 3 Biology Department, Grand Valley State University, 1 Campus Drive, Allendale, Michigan 49401, United States of America; 4 Department of Biology and Environmental Studies, Faculty of Education, Charles University in Prague, Magdalény Rettigové 4, 116 39, Prague, Czech Republic; 5 Institute of Archaeology, Faculty of History, Adam Mickiewicz University in Poznan, Umultowska 89D, 61-614 Poznan, Poland; 6 Taras Shevchenko University in Tiraspol, Taras Shevchenko University in Tiraspol, October Street 25, 33-00 Tiraspol, Moldova; 7 Institute of Archaeology, National Academy of Sciences of Ukraine, Lanzheronivska Street, 65026, Odessa, Ukraine; 8 Molecular Biology Techniques Laboratory, Faculty of Biology, Adam Mickiewicz University in Poznan, Umultowska 89, 61-614 Poznan, Poland

## Abstract

Scythians were nomadic and semi-nomadic people that ruled the Eurasian steppe during much of the first millennium BCE. While having been extensively studied by archaeology, very little is known about their genetic identity. To fill this gap, we analyzed ancient mitochondrial DNA (mtDNA) from Scythians of the North Pontic Region (NPR) and successfully retrieved 19 whole mtDNA genomes. We have identified three potential mtDNA lineage ancestries of the NPR Scythians tracing back to hunter-gatherer and nomadic populations of east and west Eurasia as well as the Neolithic farming expansion into Europe. One third of all mt lineages in our dataset belonged to subdivisions of mt haplogroup U5. A comparison of NPR Scythian mtDNA linages with other contemporaneous Scythian groups, the Saka and the Pazyryks, reveals a common mtDNA package comprised of haplogroups H/H5, U5a, A, D/D4, and F1/F2. Of these, west Eurasian lineages show a downward cline in the west-east direction while east Eurasian haplogroups display the opposite trajectory. An overall similarity in mtDNA lineages of the NPR Scythians was found with the late Bronze Age Srubnaya population of the Northern Black Sea region which supports the archaeological hypothesis suggesting Srubnaya people as ancestors of the NPR Scythians.

The Eurasian Steppe is a vast grassland region that stretches from the Carpathian foothills to Outer Mongolia. For millennia, the steppe was home to human populations that had a significant and long-lasting impact on the cultural history of the Eurasian continent. One such group that emerged at the beginning of the Iron Age had developed into an ethno-cultural agglomerate commonly referred to as the Scythians. The Scythians are best known from ancient Persian, Greek and Assyrian literary sources mainly for their nomadic warrior lifestyle, but they are also known to have actively practiced farming, pastoralism and may have been among the earliest peoples to master the art of horseback riding^1^.

The territory occupied by contemporaneous nomadic and semi-nomadic Scythian groups with shared elements of material culture extended between the lower stretches of the Danube River in the west and the Yenisei River in the east^2^,^3^ (Fig. 1). Most archaeology and history researchers suggest that the core territory of the tribes designated as “Scythians” in historic literature of Antiquity covered the steppe and forest-steppe regions of the northern Black Sea (the North Pontic region, NPR) between the lower Danube and Don rivers^4^,^5^. Together with other contemporaneous groups they formed the ‘Scythian horizon’. These groups are collectively referred to by some researchers as Scytho-Siberians, who inhabited the steppe regions to the east of NPR Scythians and included Saka from Kazakhstan (7^th^–3^rd^ century BCE)^6^,^7^ and Pazyryks (5^th^–3^rd^ century BCE) from the Sayano-Altai region of Siberia^2^,^8^. The forest-steppe zone of the core Scythian territory in the NPR was settled by populations with agro-pastoral economy, while the nomadic and semi-nomadic Scythian tribes occupied the steppe regions adjacent to the northern Black Sea. Archaeological studies of the Scythian and pre-Scythian period sites in the forest-steppe zone of the NPR pointed towards autochthonous origins of local agro-pastoral Scythian populations. Those groups which first emerged between the middle 7^th^–3^rd^ century BCE, may have been formed on the foundation of pre-existing groups belonging to such cultures as Srubnaya (Timber Grave) and Thracian Hallstatt^9^. The origins of nomadic Scythians in the steppes of Central Asia^10^ were supported by recent archaeological findings of elements of developed Scythian material culture in a series of kurgans in the western Sayan Mountains in southern Siberia dated to 9^th^–7^th^ century BCE^11^.

The relationships between the groups of the Scythian horizon are not fully understood. Some researchers regard the NPR Scythians and Scytho-Siberians as one society on account of similar animal motifs on the products of their material culture, while others consider them to be different populations each having a distinct origin and geographic specificity yet sharing cultural traditions^12^. Moreover, the ancestral relationships between the NPR Scythians and local predecessor populations in the Ponto-Caspian region are neither fully resolved.

Most of the current knowledge about the genetic relations among the populations belonging to the Scythian cultural horizon is based on studies of non-coding mitochondrial DNA (mtDNA) fragments from Scythian remains from the lower Don and southern Ural regions in Russia^13^ as well as Pazyryks from Altai and Inner Mongolia^7^,^14^,^15^,^16^,^17^,^18^. To date, only one complete mt genome of a Scythian individual from Russia has been published^19^. Mitochondrial lineages in the studied populations consist of an overlapping mix of haplogroups of east and west Eurasian descent, which does not clarify their ultimate origins. Here we aim to identify the maternal origin of the NPR Scythians and their genetic affinities to other contemporary Scythian groups through the analyses of complete mtDNA genomes. The investigated human remains were excavated from kurgans, crypts and ground burials in the main area of Scythian distribution, including territories in the lower Dniester, lower and middle Dnieper and Crimea.

## Materials and Methods

### Materials

We extracted ancient DNA (aDNA) from 29 Iron Age Scythian individuals excavated in present-day Moldova (*n* = 21) and Ukraine (*n* = 8). Moldavian samples were obtained from the Archaeological Laboratory collection at the Taras Shevchenko University in Tiraspol. Ukrainian Scythians came from the Archeology Archives of the Institute of Archaeology, National Academy of Sciences of Ukraine in Kyiv and Odessa, and the Department of Culture and Tourism of the Cherkasy Regional State Administration. Detailed information about the ancient individuals can be found in Supplementary Information Text (Materials and Methods) and Supplementary Table S1.

From each Scythian individual from Moldova two teeth were collected and transferred to the Department of Human Evolutionary Biology at the Adam Mickiewicz University in Poznań (AMU) in Poland. Ukrainian samples consisted of both bone fragments (n = 5) and teeth (n = 3), which were analyzed at the Archaeological Research Laboratory (AFL), Department of Archaeology and Classical Studies, Stockholm University (SU) in Sweden and the Archeogenetics Laboratory at Grand Valley State University (GVSU) in Allendale, Michigan, USA.

### DNA extraction, library construction and Illumina sequencing

All procedures leading to Next Generation Sequencing (NGS) were performed in the ancient DNA laboratories located at AMU and the AFL. Mitochondrial hypervariable region I (HVRI) and haplogroup-diagnostic coding regions of mtDNA of the Ukrainian samples were analyzed at GVSU using the methods described in the Supplementary Information Text (Materials and Methods). Osteological samples underwent decontamination procedures including NaOCl and UV treatments (Supplementary Information Text (Materials and Methods)), followed by DNA extraction as described by refs 20 and 21. Illumina-compatible blunt-end libraries were prepared following^22^ and screened on Illumina HiSeq 2500 High Output v4 (2 × 125 bp). For more details, see Supplementary Information Text (Materials and Methods).

Illumina sequencing was performed at the National Genomics Infrastructure (NGI) in Stockholm, Sweden. Sequence data were merged and mapped to human genome as previously published^23^. All primary pipeline computations were performed on resources provided by the Swedish National Infrastructure for Computing (SNIC) through Uppsala Multidisciplinary Center for Advanced Computational Science (UPPMAX) under projects b2013240 and b2015307^24^.

### Mitochondrial DNA capture enrichment and Ion Torrent PGM sequencing

We used commercially biotinylated probes (MYbaits^®^) for human mtDNA provided by MYcroarray^®^ (Ann Arbor, MI, USA; www.mycroarray.com) for mtDNA enrichment by hybridization capture on the Illumina sequencing libraries. The procedure was undertaken on 25 libraries, which did not yield sufficient mtDNA genome coverage (less than 5x), after initial Illumina screening (Supplementary Table S1). We performed two rounds of target enrichment following the manufacturer’s protocol version 2.3.1 (http://www.mycroarray.com/pdf/MYbaits-manual-v2.pdf). More details about mitochondrial genome capture can be found in Supplementary Information Text (Materials and Methods). Enriched and amplified indexed libraries were purified using MinElute spin columns (Qiagen) and quantified on 2200 TapeStation (Agilent Technologies, Inc.). Prior to the amplification by emulsion PCR, the indexed libraries were pooled in equimolar concentrations and adjusted to a final concentration of 20 pM^25^. Clonal template amplification on Ion Sphere Particles (ISPs) was performed using the Ion Torrent One Touch System II and the Ion One Touch 200 template kit v2 DL with regard to manufacturer’s instructions. Sequencing of the templated ISPs was conducted with the use of Ion PGM HI−Q Seq kit and Ion Torrent Personal Genome Machine (Ion PGM) system (Ion Torrent, Thermo Fisher Scientific, Inc.) at Molecular Biology Techniques Laboratory, Faculty of Biology, AMU.

### Bioinformatic analysis

Customizable analytical pipeline was used to process Illumina sequencing data as described in ref. 23. Read pairs were merged, and adapters were trimmed according to ref. 22. Merged reads were mapped as single-end reads against the revised Cambridge Reference Sequence (rCRS)^26^,^27^ (GenBank: NC_012920) with the use of BWA software package version 0.7.8^28^.

We processed sequence data from PGM Ion Torrent using a pipeline adjusted specifically to the Ion Torrent reads. Sequences were demultiplexed by barcodes using the FASTX-Toolkit (http://hannonlab.cshl.edu/fastx_toolkit/). Long (−M 110), short (−m 35), and low-quality sequences (−q 20) were removed using Cutadapt v.1.8.1^29^. The filtered sequence reads were analyzed with FastQC v 0.11.3^30^, followed by mapping against the rCRS using TMAP v3.4.1^31^. For more details, see Supplementary Information Text (Materials and Methods).

FilterUniqueSAMCons.py was used to collapse clonal reads with identical start and end coordinates, for both PGM and Illumina sequence data as in ref. 22. Misincorporation patterns were determined with the use of mapDamage v2.0.5^32^. Final sequences were visualized using Biomatters IGV software v2.3.66^33^. ANGSD v0.910^34^ was applied to build consensus sequence accepting only reads with mapping score of 30, a minimum base quality of 20, and a minimum coverage of 3.

Mitochondrial haplotypes were determined for each sample with the use of HAPLOFIND^35^, and the PhyloTree phylogenetic tree build 17^36^. The mutations reported as unexpected or missing were visually inspected in the original binary alignment map (BAM) files in IGV^33^.

The ratio of reads mapping to Y and X chromosomes (R_y_) was calculated to determine molecular sex of individuals sequenced on the Illumina platform^37^. Individuals with the R_y_ ratio ≤ 0.016 were identified as females, while those with *R*_Y_ ≥ 0.077 were determined as males (Supplementary Information Text Table S7). The molecular sex calculation was restricted only to the DNA sequence reads with mapping qualities of at least 30.

### Statistical analysis

Comparative ancient samples used in the statistical analyses were retrieved from the web depositories (NCBI Nucleotide, European Nucleotide Archive) and literature. Detailed information about comparative samples used in principal component analysis (PCA), pairwise genetic distances (*F*_ST_) and median network is shown in Supplementary Tables S2–S4.

To calculate PCA we additionally included Asian origin mtDNA haplogroups (A, B, D, F, G, M) and their frequencies to cover suggested Asian influences into Scythian populations (Supplementary Table S2). PCA was computed using RapidMiner Studio 7 (RapidMiner Inc., Boston, MA, USA) and plotted using Matplotlib 1.5.1 Python package^38^.

We have performed top-down clustering analysis in the form of k-means algorithm to explore the unbiased population relations in the PCA based on haplogropup frequencies sample-set. K-means analysis was performed in RapidMiner Studio 7 (RapidMiner Inc., Boston, MA, USA).

Pairwise genetic distances (*F*_ST_) were applied only to samples with complete ancient mt genomes (Supplementary Table S3). *F*_ST_ values and Nei’s average number of pairwise differences^39^ were computed in Arlequin 3.5^40^. We tested the hierarchical partitioning of the genetic variance in different setups by analysis of molecular variance (AMOVA)^41^ as implemented in Arlequin. Populations used in AMOVA were the same as for the *F*_ST_ analysis and the list is provided in Supplementary Table S3. We have tested several configurations of population groupings to evaluate the best matching position of Scythian population from the NPR and its relation to other populations. Multidimensional scaling (MDS) of *F*_ST_ values was computed using Python scikit-learn 0.17 package^42^. The maps were created using QGIS 2.12.2^43^.

The median network was calculated and computed for the haplogroup U5 using the Networks 4.614 software (fluxus-engineering.com) with the most common mutations weighted reversely to their frequency.

## Results

We successfully retrieved complete mt genomes for 19 out of the 29 Scythian individuals (Table 1 and Supplementary Table S1). Shotgun screening of genomic DNA libraries generated mt genomes for four individuals with the coverage ranging from 6.5x to 70x. For the remaining samples, the mt capture enrichment followed by Ion Torrent sequencing, provided additional 15 complete mt genomes with the depth of coverage from 7x to 103.9x. Mitochondrial DNA genome data were deposited in GenBank under accession numbers KX977302-KX977320.

The analysis of damage patterns revealed the presence of damages typical for aDNA, i.e. C-T and G-A transitions accumulated at 5′ and 3′ ends, respectively (Supplementary Fig. S11).

Mitochondrial lineages in the NPR Scythians analyzed in this study appear to consist of a mixture of west and east Eurasian haplogroups. West Eurasian lineages were represented by subdivisions of haplogroup U5 (U5a2a1, U5a1a1, U5a1a2b, U5a2b, U5a1b, U5b2a1a2, six individuals total, 31.6%), H (H and H5b, three individuals total, 15.8%), J (J1c2 and J2b1a6, two individuals, 10.5%), as well as haplogroups N1b1a, W3a and T2b (one individual each, 5.3% each specimen). East Eurasian mt lineages were represented by haplogroups A, D4j2, F1b, M10a1a1a, and H8c (represented by a single individual), in total, comprising 26.3% of our sample set.

The results of the low-resolution mtDNA screening at GVSU were consistent with whole mt capture results from AMU (see Supplementary Table S1). Polymorphism patterns uncovered in the coding and HVRI regions in specimens SCY009 and SCY011 identified their lineages to belong to haplogroups J and A respectively. Specimens SCY006 and SCY010 were assigned to the M* and N* clades respectfully.

**Figure 2:**
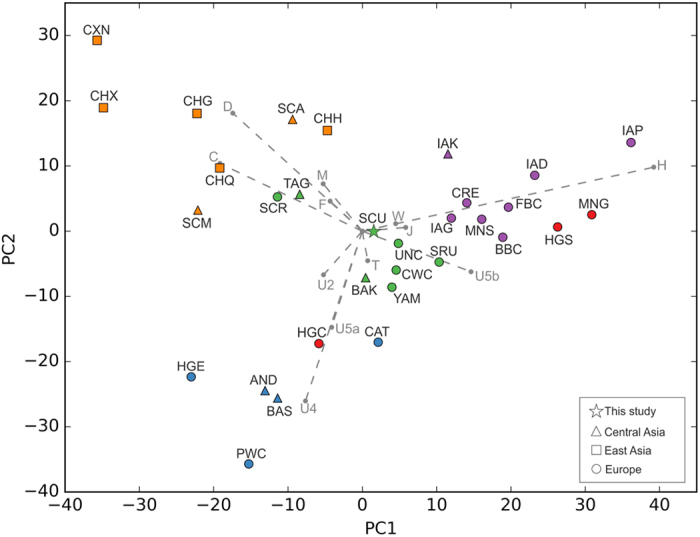
PC analysis based on mitochondrial DNA haplogroup frequencies. The two dimensions account for 43.4% of the total variance. Haplogroup contributions are represented by loading vectors marked on the plot as grey arrows. Populations are grouped into five clusters according to k-means. First cluster (in green): BAK, Bronze Age Kazakhstan; CWC, Corded Ware culture; SCR, Scythians from Russia; SCU, Scythians from Moldova and Ukraine, present study; SRU, Srubnaya culture; TAG, Tagar culture; UNC, Unetice culture; YAM, Yamnaya culture. Second cluster (in red): HGC, Hunter-Gatherers Central Europe; HGS, Hunter-Gatherers Southern Europe; MNG, Middle Neolithic Germany. Third cluster (in blue): AND, Andronovo culture; BAS, Bronze Age Siberia; CAT, Catacomb culture; HE, Hunter-Gatherers East Europe; PWC, Pitted Ware culture. Fourth cluster (in purple): BBC, Bell Beaker culture; CRE, Crete Minoans; FBC, Funnel Beaker culture; IAD, Iron Age Denmark; IAG, Iron Age Germany; IAK, Iron Age Kazakhstan; IAP, Iron Age Poland; MNS, Middle Neolithic Southern Europe. Fifth cluster (in orange): CHH, Western Hun, China; CHG, Gavaerg China; CHQ, Quin to Western Jin, China; CHX, Xiaohe, Xinjiang, China; CXN, Xiongnu, Mongolia; SCA, Scytho-Siberians from Altai; SCM, Pazyryk culture from Mongolia. Detailed descriptions and references of comparative populations are listed in Supplementary Table S2.

To trace genetic affinities between Scythians from present day Moldova and Ukraine (SCU) and other European and Asian ancient populations, their mt haplogroup frequencies were visualized in the space of principle components. The PCA plot of the first two components accounted for 43.4% of the total variance (Fig. 2). The SCU group was located in direct proximity to the central European Neolithic Corded Ware culture (CWC). It also grouped near a number of Bronze Age populations from eastern and central Europe (Srubnaya (SRU), Yamnaya (YAM) and Unetice (UNC)), as well as from central Asia (Bronze Age Kazakhstan (BAK)). Finally, k-means clustering (k value = 5), grouped SCU within a cluster further encompassing Scythians from Russia (SCR) and Tagar culture from southern Siberia (TAG). The Pazyryks from Mongolia (SCM) and Altai (SCA) were separated from SCU and grouped within the k-mean cluster consisting of Central and East Asian populations.

**Figure 3:**
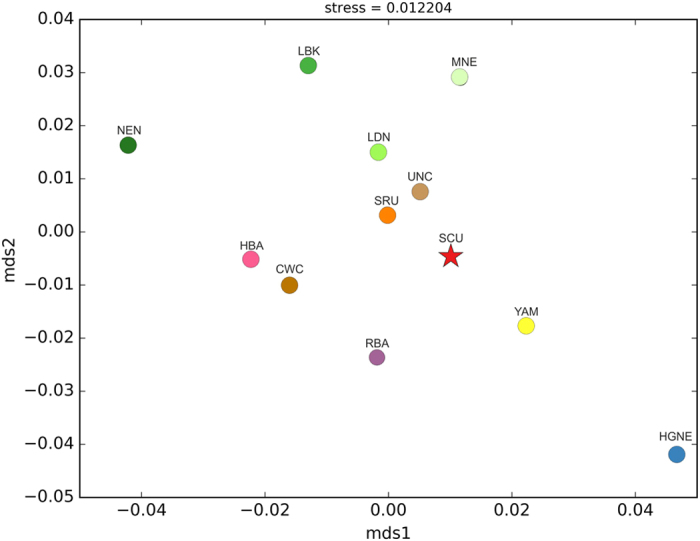
MDS plot based on *F*_ST_ calculated from complete mitochondrial genomes. Population abbreviations: CWC, Corded Ware culture; HBA, Bronze Age populations from the geographic area of modern Hungary including Maros, Vatya, Baden, Kyjatice cultures; HGNE, combined Hunter-Gatherers from the North and East; LBK, Linear Pottery culture; LDN, Late Danubian cultures; MNE, Middle Neolithic cultures; NEN, Near Eastern Neolithic; RBA, Bronze Age populations from the geographic area of present-day Russia including Afanasievo, Andronovo, Poltavka, Potapovka and Sintastha individuals; SRU, Srubnaya culture; UNC, Unetice culture; YAM, Yamnaya culture; SCU, Scythians from Moldova and Ukraine from present study combined with one individual from Rostov-on-Don^59^. Detailed information about each individual from particular comparative population is provided in Supplementary Table S3.

**Figure 4:**
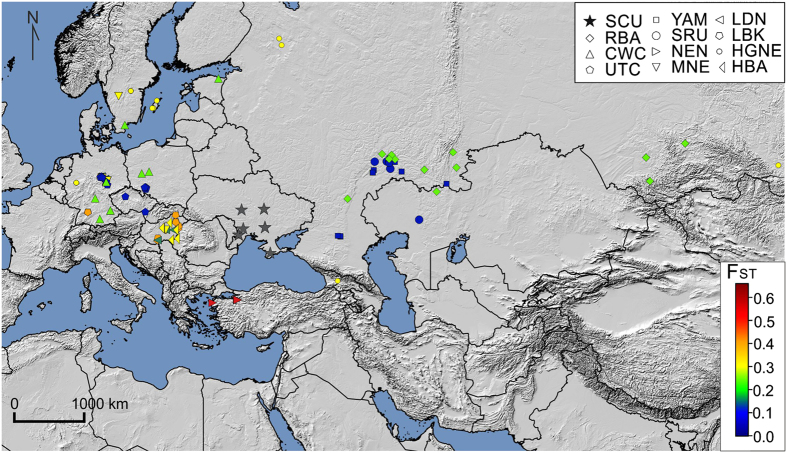
Heatmap of *F*_ST_ and geographic distribution. Colors in gradient reflect *F*_ST_ values. Population abbreviations are as in Fig. 3. The map was created using QGIS 2.12.2^43^.

Slatkin’s linearized pairwise *F*_ST_ values calculated on complete mt genomes and visualized using MDS (Fig. 3) and heatmap (Fig. 4) indicate that the studied Scythian samples are closest to the Srubnaya (SRU) (*F*_ST_ = 0.00; p < 0.05) and Yamnaya (YAM) (*F*_ST_ = 0.006; p < 0.05) populations (see Supplementary Table S5), followed by Unetice (UNC) (*F*_ST_ = 0.008; p < 0.05) and Corded Ware Culture (CWC) (*F*_ST_ = 0.023; p < 0.05). The European Neolithic Linear Ware culture (LBK) (*F*_ST_ = 0.043; p > 0.05) and Near Eastern Neolithic populations (NEN) (*F*_ST_ = 0.062; p > 0.05) appeared most distant to the individuals in our dataset. Correspondingly, in the resulted *F*_ST_ based MDS plot (stress value of 0.012), our Scythian group positioned proximately to Srubnaya (SRU), Yamnaya (YAM) and Unetice (UNC) populations and distantly to Near Eastern and European Neolithic (NEN, LBK) and Near Eastern hunter-gatherer (HGNE) populations (Fig. 3).

Analysis of molecular variance (AMOVA) summarized and tested the distribution of genetic variability within and between subpopulations. We tested several other combinations of grouping Scythians with two or more populations. The results suggested the combination with the highest intragroup and lowest intergroup genetic variability to be the Scythians-Srubnaya sample-set (4.93% of variability among groups, −0.73% among populations within groups). Second best result was observed in combination of Scythians with Unetice sample-set (3.05% among groups, 1.14% among populations). The AMOVA results are summarized in Supplementary Table S6.

**Figure 5:**
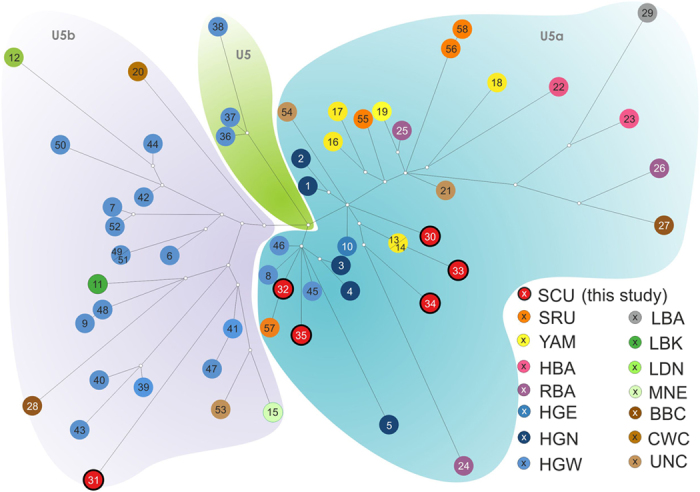
Median network of U5 haplotypes. Colors of nodes represent archaeological cultures where a particular haplotype was found. Numbers in nodes indicate particular haplotype (1–58) summarized in Supplementary Table S4. Population abbreviations: CWC, Corded Ware culture; BBC, Bell Beaker culture; HBA, Bronze Age populations from the geographic area of modern Hungary including Maros and Vatya individuals; HGE, Hunter-Gatherers from the East; HGN, Hunter-Gatherers from the North; HGW, Hunter-Gatherers from the West; LBK, Linear Pottery culture; LDN, Late Danubian cultures including a Rössen individual; MNE, Middle Neolithic cultures; NEN, Near Eastern Neolithic; RBA, Bronze Age populations from the geographic area of present-day Russia including Afanasievo and Karasuk individuals; SRU, Srubnaya culture; UNC, Unetice culture; YAM, Yamnaya culture; SCU, Scythians from Moldova and Ukraine from present study.

Considering the high number of Scythian individuals identified as belonging to haplogroup U5 (31.6%), we conducted median network analysis of published ancient haplotypes belonging to U5a and U5b subdivisions and ranging from the Mesolithic (excluding hunter-gatherers predating last glacial maximum) to the Iron Age (Fig. 5). Haplotypes used in the median network analysis are described in detail in Supplementary Table S4. The Scythians in the U5a cluster were located centrally in the median network, near the U5a ancestral node. The U5a Scythian samples (SCU) grouped together with Mesolithic hunter-gatherers from Sweden (haplotypes 3, 4 and 5), Germany (8), Russia (10) and France (45 and 46). Non-Mesolithic samples in those clusters were less numerous and included Srubnaya (SRU) (57) and Yamnaya (YAM) (13 and 14) from Samara region and one representative of Karasuk culture from the Altai territory (central-south Siberia) (24). A single U5b Scythian individual (31) was distant from the East European populations and was placed basally to a broader cluster composed of west hunter-gatherers (HGW) from France (41) and Germany (47) as well as chronologically younger samples from the Middle Neolithic (MNE) and Unetice (UNC) (both from Germany) (15 and 53, respectively).

## Discussion

On the basis of published data concerning the phylogeography of mt lineages distribution in ancient populations of Europe and Asia, the 19 complete mt genomes of the NPR Iron Age Scythians produced in this study fall into three main groups of different ancestry. The first group of mt lineages is represented by U5 haplotypes that are considered to be a European Hunter-Gatherer genetic component^44^,^45^. The second group comprises haplotypes belonging to H, J, T, W and N1b, ultimately connected to the genetic package of the early Neolithic farmers^44^,^46^,^47^, and the third group includes A, D, M10 and F mt lineages considered to be of East Eurasian origin^48^,^49^,^50^,^51^.

Representatives of the U5 haplogroup account for one third of the mt haplotypes identified in analyzed Scythians. These ultimately relate to West Eurasian hunter-gatherers, whose descendants extended throughout the European subcontinent and into East Eurasia from the Mesolithic to the Bronze Age. We have combined the known diversity of the prehistoric U5 lineage from different sources^19^,^45^,^46^,^47^,^52^,^53^,^54^,^55^,^56^ into a median network, which allowed for identification of candidate source populations contributing to the U5a diversity in NPR Scythians. Although the network approach is still limited by the small number of available samples, our results indicate that the source populations with the most closely related haplotypes are the Bronze Age Srubnaya (SRU) (1900-1200 BCE) and the earlier Yamnaya (YAM) (3300-2700 BCE) from the Ponto-Caspian region and Bronze Age populations from the Altai Mountains, such as Karasuk (1500-800 BCE). Diverse U5a-carrying populations of the steppe such as Yamnaya, Srubnaya and Scythians shared the nomadic lifestyle, with the economic foundations transforming from wild game exploitation in the Mesolithic to pastoralic animal husbandry in the Bronze Age. Furthermore, both Yamnaya and Srubnaya were part of the Kurgan culture phenomenon^57^ with the Scythian cultural horizon being the most representative for the kurgan building tradition.

The second group of identified mt genomes in the NPR Scythians is comprised of lineages that suggest associations of the NPR Scythians with the Neolithic European farming groups. Although the mt lineage composition of analyzed Scythians significantly differs from that seen in NEN and LBK groups (*F*_ST_ = 0.06, *F*_ST_ = 0.04, respectively, p > 0.05), particular lineages such as J1c2, T2b, H/H5, H, W and N1b1a ultimately go back to the earliest European farmers. Lineages of J1c, H5 and T2b belong to the Neolithic farming package of mtDNA haplogroups which have been found in most Neolithic and Bronze Age European populations^19^,^44^,^47^,^55^,^58^. Noteworthy, individuals belonging to the N1b1a have been found in the Neolithic Anatolia^19^,^59^, but the lineage has not appeared in any other Eurasian Neolithic, Bronze or Iron Age populations to date. Therefore, the presence of N1b1a in Scythians could be attributed to population migrations along the southern boundaries of the Ponto-Caspian region^60^.

Mitochondrial lineages T2b, W3a and J2b1a6 have been identified in representatives of the Bronze Age groups such as Srubnaya (T2b, J2b1a, H5)^19^, Sintashta (J2b1a, J1c)^55^, Yamnaya (W3a and T2b)^47^,^61^ and Mezhovskaya (J2b1a)^55^ along with East European Eneolithic Trypillian culture (T2b)^62^, suggesting genetic continuity of these lineages from at least the Bronze Age or even Neolithic times in NPR region. This is further supported by published data^19^ which showed that almost one fifth of the genetic makeup of the Late Bronze Age Srubnaya people of the Ponto-Caspian region is of the Early European Farmer or Anatolian Neolithic ancestry possibly resulting from the admixture of populations related to Early European Farmers and Yamnaya. Thus, if the NPR Scythians are the descendants of populations related to Srubnaya, the origin of the identified farming lineages would likely be within the steppe/forest-steppe region between the NPR and southern Ural. According to previous genomic studies^47^,^55^ the CWC people are likely to have arisen from Yamnaya background. Thus, genetic affinities of the NPR Scythians to the Yamnaya people might also explain their close genetic similarity to the CWC reflected in PCA and *F*_ST_ results. Close genetic relations of the NPR Scythians and Srubnaya are supported by the *F*_ST_ analyses revealing no significant differences between these two populations (Figs 3 and 4, Supplementary Table S5). AMOVA further corroborated these results showing that the combination of Scythians and Srubnaya results in the lowest genetic variability among populations in a group and the highest variability between groups (Supplementary Table S6). Our results support the archaeological interpretations concerning the origin of the Scythians. One of the versions of this theory suggests that the Srubnaya people have migrated in several waves from the Volga-Ural region to the NPR during the second half of the second millennium BCE where their descendants gave rise to the Scythians around the 7^th^ century BCE^63^.

The third group of lineages identified in the NPR Scythians is derived from East Eurasian ancestry. Since, to our knowledge, there is no evidence of agricultural subsistence in East Eurasian Scythians, these lineages should be considered to be genetic components associated with nomadic populations. Mitochondrial haplogroups such as A, D and F have already been found in samples from the Mesolithic, Neolithic, Bronze and Iron Age southern Siberia and the Altai^7^,^14^,^15^,^16^,^18^,^55^,^64^,^65^,^66^,^67^. Notably, haplogroup M10 found among Scythians from Glinoe, is present not only in far East Asia but also in modern populations of the Altai^68^. Among ancient populations, the M10 lineages have been found in Chinese specimens from southern Xinjiang^48^ (8^th^ -1^st^ century BCE) and Xiongnu^69^ (3^rd^ century BCE). Furthermore, haplogroup M have been identified in Pleistocene individuals from western Europe but it is thought that these lineages disappeared due to bottleneck effect in the Last Glacial Maximum^56^. Haplogroup H8c, identified in the NPR Scythians likely belongs to East Eurasian lineages as well. Sequence analysis of 830 modern Eurasian mt genomes suggested a distinct phylogeographc history for H8, with a clustering of Near Eastern and Central Asian haplotypes of H8 and a pronounced presence of carriers of H8c in the Altai region^70^. It was further hypothesized that the distribution of this lineage could have been facilitated by nomadic migrations along the NPR coast^70^. Although our PCA analysis showed Altai Pazyryk (SCA) to be distant from the NPR Scythians (SCU) (Fig. 2), it must be emphasized that the presence of haplogroups A and H8c in the analyzed population connects NPR Scythians to the Altai and identifies this region as a possible source of these East Eurasian mt lineages. The only Scythian mt genome from southern Urals published thus far also belonged to an East Eurasian lineage, G2a^19^. The Y chromosomal lineage (R1a1a1b2a2a) reconstructed for this individual was supposedly characteristic for members of the Srubnaya culture^19^ which additionally supports our conclusions concerning close genetic links between Scythians and people related to the late Bronze Age Srubnaya.

Previous analyses of mtDNA HVRI sequence data from Scythians inhabiting Rostov-on-Don region in eastern NPR also resulted in the identification of East Eurasian lineages, such as D^13^. Moreover, mt lineage D4j2 identified in the individual SCY006 was shown to have been dominant in the Pazyryk culture from Altai and Inner Mongolia (4^th^ -2^nd^ century BCE)^7^,^14^,^16^,^17^,^18^. The presence of East Eurasian mt lineages supports those archaeological theories that acknowledge the influence of an East Eurasian element in the formation of the Scythian horizon^71^,^72^.

Scythians from Rostov-on-Don as well as Pazyryks from Altai and Inner Mongolia were carriers of mixed east and west Eurasian lineages, with the dominant presence of the latter at 62.5% and 53.3%, respectively^7^,^13^,^14^,^15^,^16^,^17^,^18^. Mitochondrial haplogroup analyses of the NPR Scythians from this study and those from Rostov-on-Don and Pazyryks from Altai and Inner Mongolia, reveal that, for the most part, the same lineages are found in all three groups and are often singularly represented in each group. Noteworthy, comparing the frequencies of east and west Eurasian haplogroups in all three groups of the Scythian horizon, an east-west mtDNA lineage cline is visible, for east Eurasian lineages going west-east is from 26.3% (in present study) through 37.5% (in Scythians from Rostov-on-Don) to 46.7% (in Pazyryks) with the opposite trend for west Eurasian lineages. Otherwise, mt lineage composition is comparable in all three groups of the Scythian horizon which supports their shared maternal genetic roots founded on the common east and west Eurasian substrate with an input from neighboring populations. The genetic influx of East Eurasian haplotypes might be the result of establishing relationships between migrants with European ancestry and women of east Eurasian origin as was previously proposed by^66^ in case of Iron Age south Siberian populations. However, more detailed studies of autosomal DNA are needed to clearly resolve this issue.

## Conclusions

Sequence data from whole mt genomes indicate three potential mtDNA lineage ancestries of the NPR Scythians. The first component traces back to west Eurasian hunter-gatherers and is represented by the lineages belonging to subdivisions of haplogroup U5. The second component is composed of mt lineages connected with Neolithic farming expansion into Europe (H, J, T, W and N1b). The last ancestral mt lineage component is comprised of east Eurasian haplotypes belonging to D, A, F1, H8 and M10 which point to association of the NPR Scythians with east Eurasian populations, in particular from the Altai region. A comparison of NPR Scythian mtDNA lineages with other ancient groups suggests close genetic affinities with representatives of the Bronze Age Srubnaya population, which is in agreement with the archaeological hypothesis suggesting Srubnaya people as the ancestors of the NPR Scythians. However, to provide additional genetic support for this hypothesis data from nuclear genomes are needed.

## Additional Information

**How to cite this article:** Juras, A. *et al*. Diverse origin of mitochondrial lineages in Iron Age Black Sea Scythians. *Sci. Rep.* **7**, 43950; doi: 10.1038/srep43950 (2017).

**Publisher's note:** Springer Nature remains neutral with regard to jurisdictional claims in published maps and institutional affiliations.

## Supplementary Material

Supplementary Information

Supplementary Dataset 1

## Figures and Tables

**Figure 1:**
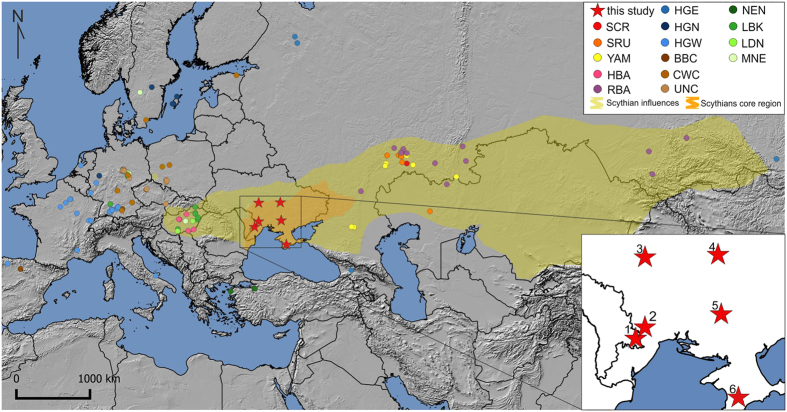
Map of the Scythian core region and territories of their possible influences in 7^th^–3^rd^ century BCE. Locations of sites investigated are marked as red stars: Glinoe (1), Vapnyarka (2), Nesterivka (3), Svetlovodsk (4), Starosillya (5), Simferopol (6). Colored circles show locations of comparative ancient populations. Population abbreviations: SCR, Scythians from Russia; SRU, Srubnaya culture; YAM, Yamnaya culture; HBA, Bronze Age populations from Hungary; RBA, Bronze Age populations from Russia; HGE, Hunter-Gatherers Eastern; HGN, Hunter-Gatherers Northern; HGW, Hunter-Gatherer Western; BBC, Bell Beaker culture; CWC, Corded Ware culture; UNC, Unetice culture; NEN, Near Eastern Neolithic; LBK, Linear Pottery culture; LDN, Late Danubian cultures; MNE, Middle Neolithic cultures. The map was created using QGIS 2.12.2^43^.

**Table 1:** Description of studied individuals.

Sample	Archaeological site	Dating of the sites based on typochronology	Age at death	Molecular sex	mtDNA haplotype
SCY192	Glinoe, Moldova	4th-2nd BCE	15–20 yrs	XX	H8c
SCY193	Glinoe, Moldova	4th-2nd BCE	n.a.	XY	U5a2a1
SCY196	Glinoe, Moldova	4th-2nd BCE	10–11 yrs	n.r.	W3a
SCY197	Glinoe, Moldova	4th-2nd BCE	n.a.	XY	U5a1a1
SCY300	Glinoe, Moldova	4th-2nd BCE	35–50 yrs	XX	H5b
SCY303	Glinoe, Moldova	4th-2nd BCE	6–7 yrs	XX	U5a1a2b
SCY305	Glinoe, Moldova	4th-2nd BCE	50 + yrs	n.r.	U5a2b
SCY308	Glinoe, Moldova	4th-2nd BCE	20–35 yrs	n.r.	F1d
SCY311	Glinoe, Moldova	4th-2nd BCE	35–50 yrs	XX	T2b
SCY332	Glinoe, Moldova	4th-2nd BCE	30–35 yrs	XX	M10a1a1a
SCY334	Glinoe, Moldova	4th-2nd BCE	20–35 yrs	n.r.	H5b
SCY001	Svetlovodsk, Ukraine	4th BCE	n.a.	n.r.	U5a1b
SCY002	Svetlovodsk, Ukraine	4th BCE	n.a.	n.r.	J1c2
SCY006	Starosillya, Ukraine	7th BCE	n.a.	XX	D4j2
SCY011	Nesterivka, Ukraine	4th BCE	n.a.	XX	A
SCY012	Vapnyarka, Ukraine	4th-3rd BCE	n.a.	n.r.	U5b2a1a2
SCY009	Starosillya, Ukraine	7th BCE	n.a.	XY	J2b1a6
SCY010	Starosillya, Ukraine	7th BCE	n.a.	XX	N1b1a
SCY005	Simferopol, Ukraine	3rd-2nd BCE	n.a.	XX	H

n.a. - not available. n.r. - no result.
