# Supplementary material for: Diverse origin of mitochondrial lineages in Iron Age Black Sea Scythians
Source: Sci Rep. 2017 Mar 7;7:43950. doi: 10.1038/srep43950 (PMC5339713; doi:10.1038/srep43950)
Supplement: Supplementary Information [file srep43950-s1.pdf]

Supplementary Information Text

Diverse origin of mitochondrial lineages in Iron Age Black Sea Scythians

Anna Juras[1*], Maja Krzewińska[2], Alexey G. Nikitin[3*], Edvard Ehler[1,4], Maciej Chyleński[5], Sylwia Łukasik[1], Marta Krenz-Niedbała[1], Vitaly Sinika[6], Janusz Piontek[1], Svetlana Ivanova[7], Miroslawa Dabert[8], Anders Götherström[2]

[1] Department of Human Evolutionary Biology, Institute of Anthropology, Faculty of Biology, Adam Mickiewicz University in Poznan, Umultowska 89, 61-614 Poznan, Poland

[2] Department of Archaeology and Classical Studies, Stockholm University Wallenberglaboratoriet, SE-106 91 Stockholm, Sweden

[3] Biology Department, Grand Valley State University, 1 Campus Drive, Allendale, Michigan 49401, United States of America

[4] Department of Biology and Environmental Studies, Faculty of Education, Charles University in Prague, Magdalény Rettigové 4, 116 39, Prague, Czech Republic

[5] Institute of Prehistory, Faculty of History, Adam Mickiewicz University in Poznan, Umultowska 89D, 61-614 Poznan, Poland

[6] Archaeological Research Laboratory, Taras Shevchenko University in Tiraspol, October Street 25, 33-00 Tiraspol, Moldova

[7] Institute of Archaeology, National Academy of Sciences of Ukraine, Lanzheronivska Street, 65026, Odessa, Ukraine Odessa, Ukraine

[8] Molecular Biology Techniques Laboratory, Faculty of Biology, Adam Mickiewicz University in Poznan, Umultowska 89, 61-614 Poznan, Poland

*** Corresponding authors:** Anna Juras, PhD, tel.: +48 61 829 56 25, fax: +48 61 829 57 30, e-mail: annaj@amu.edu.pl; Alexey G, Nikitin, PhD, tel.: +1-616-331-2505, fax: +1-616-331-3446, e-mail: nikitin@gvsu.edu

Supplementary Information Text (Materials and Methods)

Materials and archaeological context

The skeletons recovered from present-day Moldova were obtained during the excavations of 113 Scythian barrows dated from the end of the 4th century BCE to the 2nd century BCE, conducted in 1995-2015 at Glinoe site, located on the left bank of the Lower Dniester in south-eastern part of Moldova (46°66'84"N, 29°80'01"E). The chronology of the cemetery is based on burial inventory found during the excavations (mainly amphorae and epigraphic data, as well as ceramics and lamps). The majority of Scythian barrows included single graves, rarely double graves, while multiple graves occurred less frequently (Supplementary Figs S1-10). Human skeletal remains were deposited at the depth of 1-6 m below the ground level[1].

Scythian specimens SCY001, SCY002 and SCY005 came from the excavations in the Crimean Peninsula of a kurgan group (SCY001) and a ground cemetery (SC002) in Svetlovodsk in 1980 (4th c. BCE[2]) and a ground (crypt) cemetery 0.8 km from the Scythian Neapolis, the capital of Scythia Minor in the present-day Simferopol, in 1990 (SCY005, 3rd-2nd century BCE)[3]. Scythian specimens SCY006 and SCY009-011 from mainland Ukraine included kurgan groups from Starosyllya, Kherson District (SCY006, SCY009, SCY010, 7th century BCE) and Nesterivka, Cherkasy District (dated to 4th century BCE). Scythian specimen SCY012 came from an Eneolithic-Bronze Age kurgan near the village of Vapnyarka in the Odessa District of Ukraine in western NPR, ~100 km southeast from Glinoe, excavated in 2008 and dated to 4th-3rd c. BCE[4].

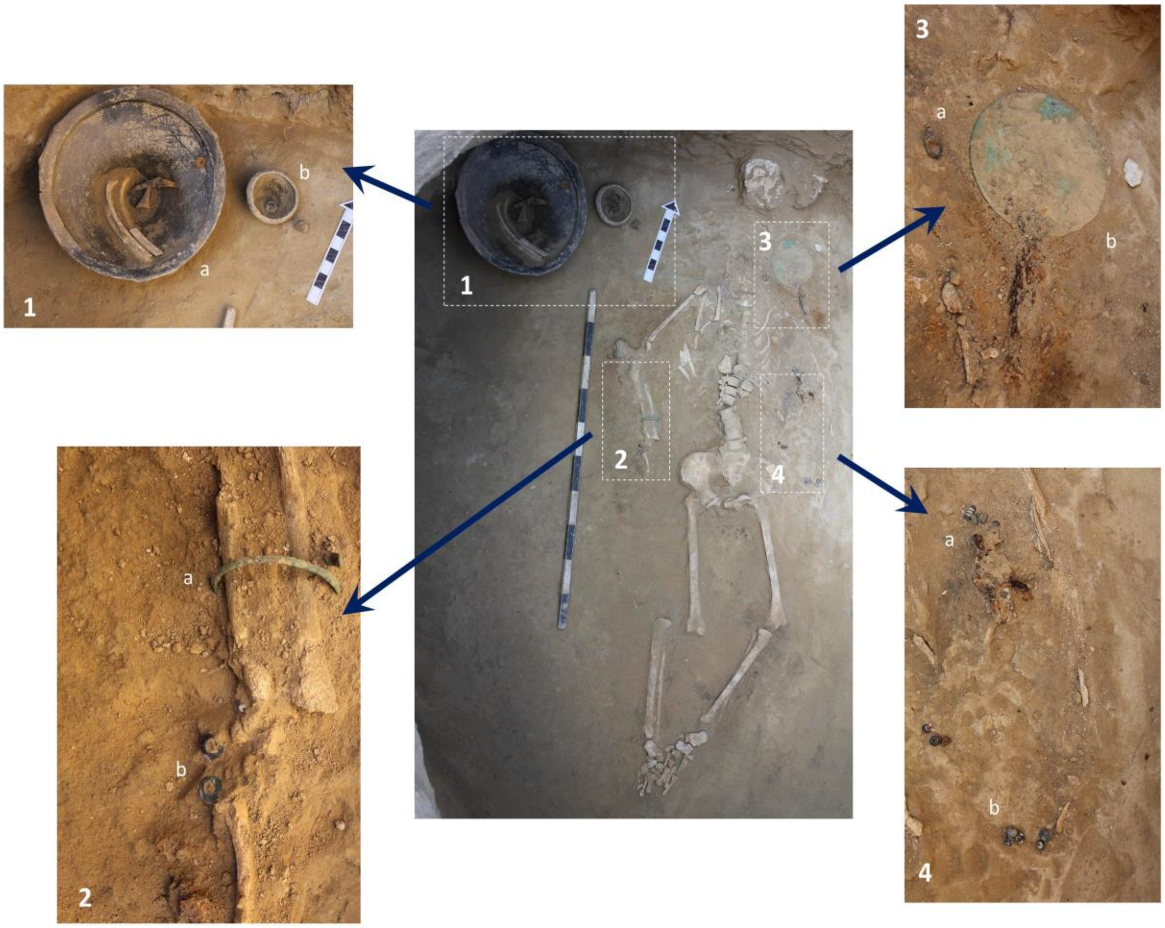

Supplementary Fig S1. Barrow 103 burial 1, Glinoe site, Moldova (SCY192); 1a – handmade cup with iron knife and animal bones; 1b – handmade bowl with 2 spindle whorls (inside and near it); 2a – bronze bracelet; 2b – glass beads, 3a – silver earring; 3b – bronze mirror with iron handle, 4a – glass beads; 4b – glass beads.

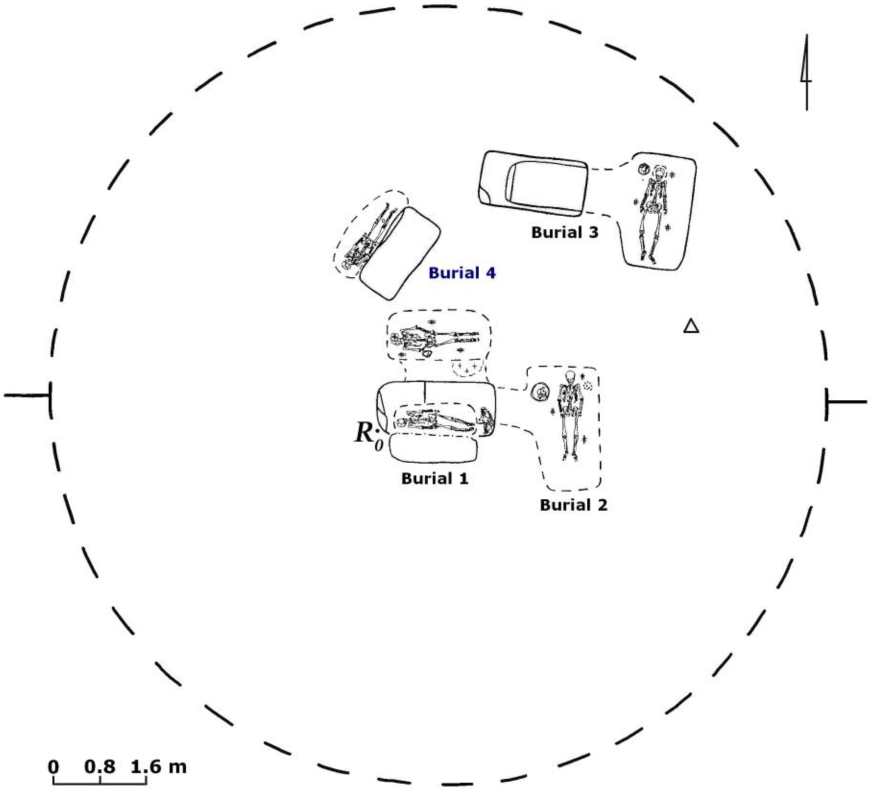

Supplementary Fig S2. Barrow no 22, Glinoe site, Moldova; burial 4 with SCY196 is in blue.

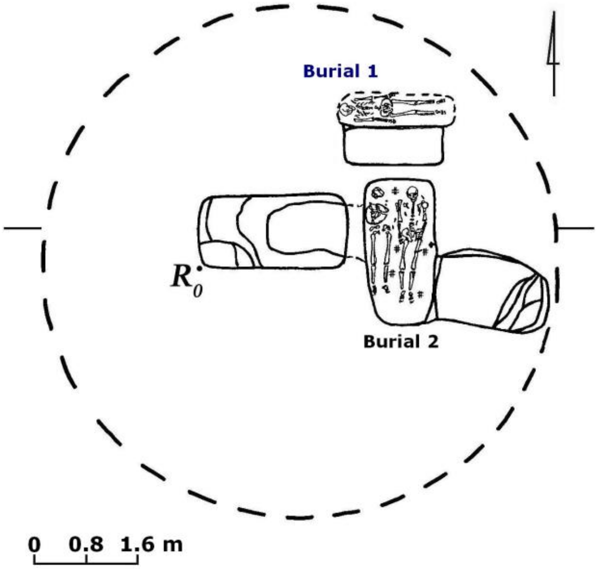

Supplementary Fig S3. Barrow no 43, Glinoe site, Moldova; burial 1 with SCY311 is in blue.

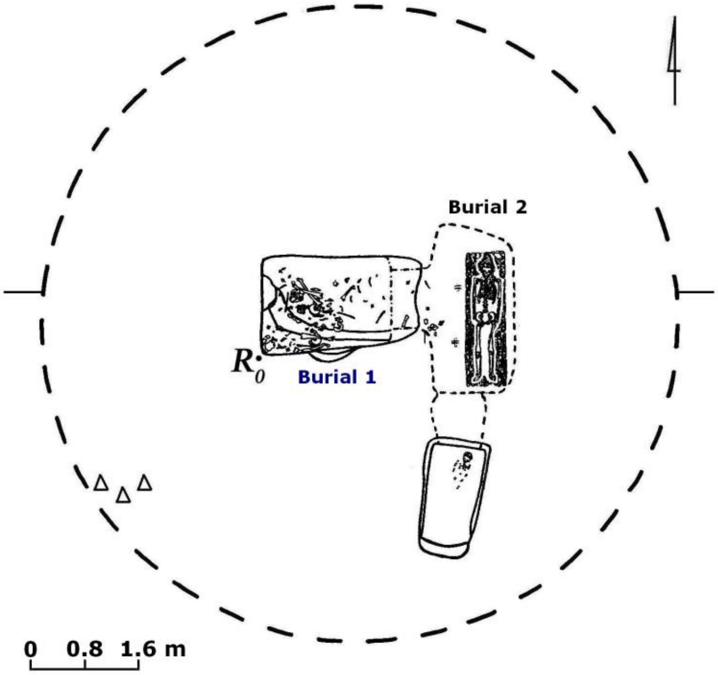

Supplementary Fig S4. Barrow no 50, Glinoe site, Moldova; burial 1 with SCY197 is in blue.

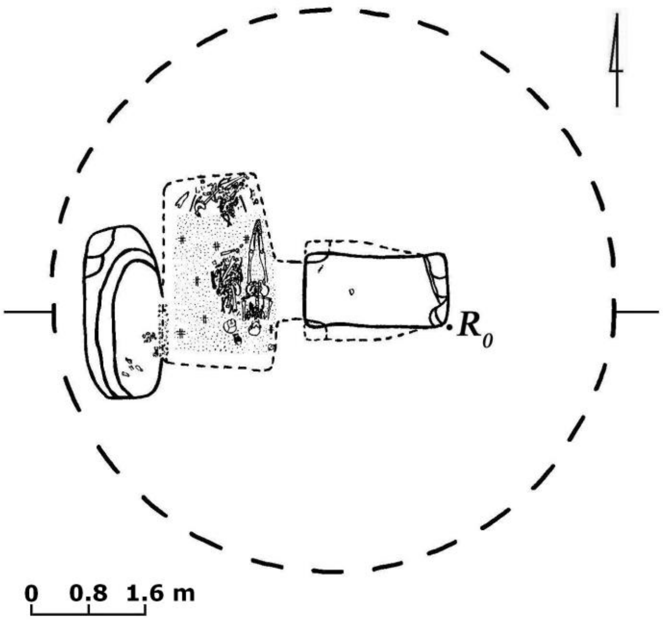

Supplementary Fig 5. Barrow no 53, Glinoe site, Moldova; human remains of SCY308.

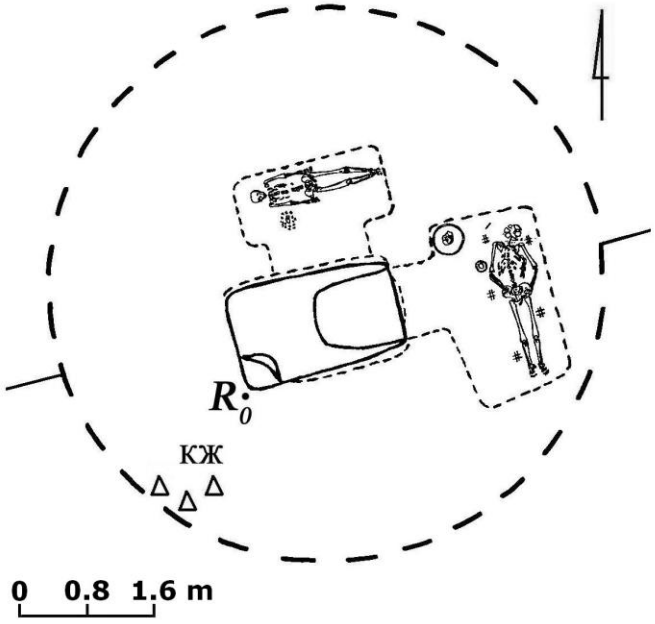

Supplementary Fig S6. Barrow no 65, Glinoe site, Moldova; human remains of SCY332.

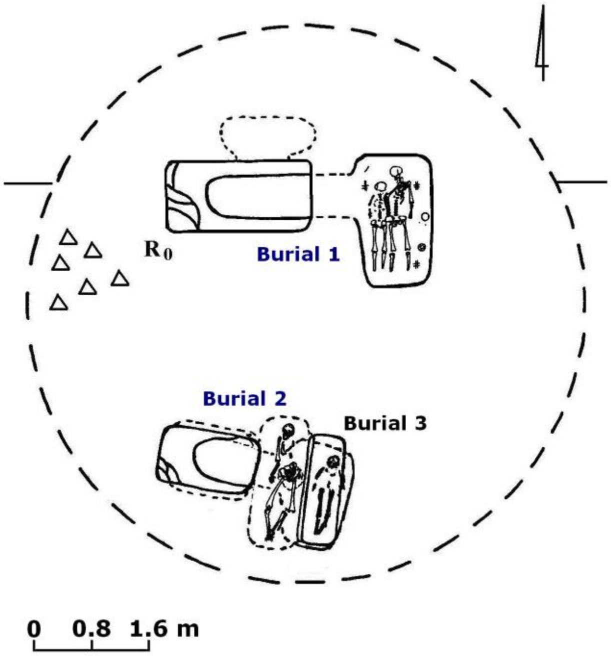

Supplementary Fig S7. Barrow no 75, Glinoe site, Moldova, burial 1 with SCY193 and burial 2 with SCY303 are in blue.

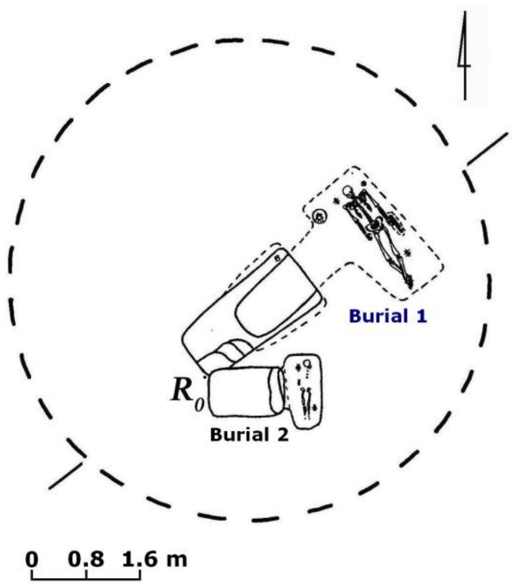

Supplementary Fig S8. Barrow no 87, Glinoe site, Moldova; burial 1 with SCY305 is in blue.

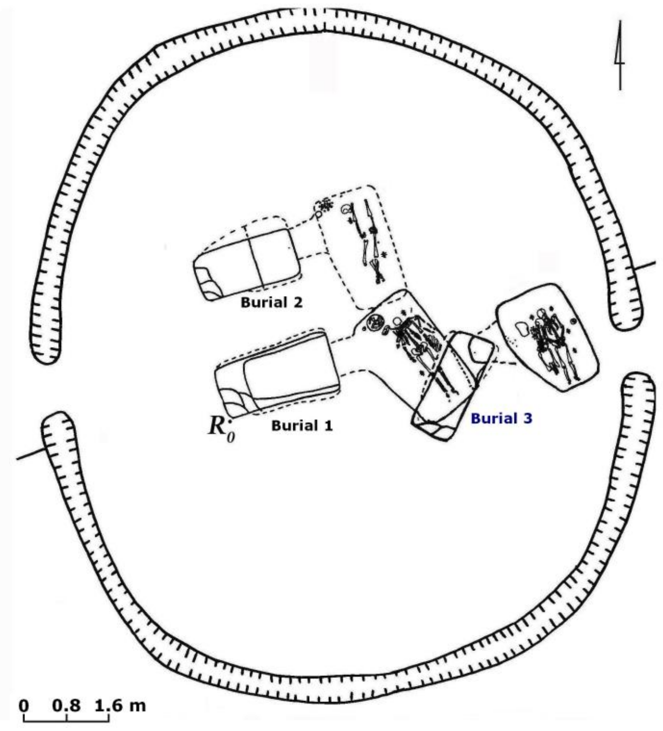

Supplementary Fig S9. Barrow no 89, Glinoe site, Moldova; burial 3 with SCY334 and SCY300 is in blue.

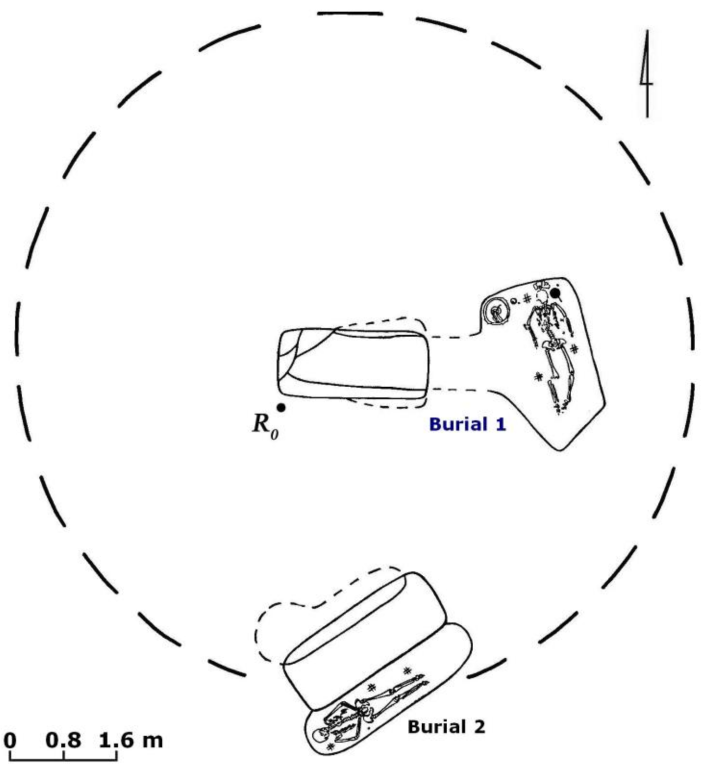

Supplementary Fig S10. Barrow no 103, Glinoe site, Moldova; burial 1 with SCY192 is in blue.

References:

1. Telnov, N, Chetvernikov, I, Sinika, Skifskiy mogilnik III-II vv. do n. e. u s. Glinoye na levoberezh'ye Nizhnego Dnestra (predvaritel'nyye itogi issledovaniya). *Drevnosti severnogo Prichernomorya III-II w. do n.e. Mezhdunarodnaya Nauchnaya Konferentsiya 16-17 okt. Tiraspol* (ed. Telnov, N.) 5-15 (PSU, 2012).
2. Bokij, N. M. Pozdneskifskij bezkurgannyj mogil'nik u g. Svetlovodska. Arkheologicheskie issledovanija na Ukrainye v 1978-1979 gg. in *Tezisy dokladov XVIII Konferentsii IA AN USSR* 101 (Dnepropetrovsk, 1980).
3. Puzdrovsky, A.E. Gruntovyj sklep rubezha n.e. iz okrestnostej Neapolya Skifskogo, in *Severnoe Prichernomor'e v antichnoe vremya* (ed. Puzdrovsky, A.E.) 162–172 (Kiev, 2002).
4. Ivanova, S. V. Novi radiovugletsevi daty dlya pam'yatok Pivnichno-Zakhidnogo Prichornomor'ja. *Arheologia* **3,** 69–75 (2010).

Ancient DNA analysis - Adam Mickiewicz University (AMU) and Archaeological Research Laboratory (AFL), Stockholm University

DNA extraction and screening

DNA extraction from Moldova samples (*n*=21) was performed in sterile ancient DNA laboratory at the Adam Mickiewicz University (AMU) in Poland. The surface of the teeth was cleaned with 0.5-5% NaOCl and rinsed with sterile water, followed by UV exposure (254 nm) for two hours per each site. After UV irradiation, roots of teeth were drilled using Dremel® drill bits and bone powder (~250 mg) was collected to sterile screw cap tubes (2 ml). DNA was extracted using protocols presented by[1] and[2] DNA extract (20 µl) was used to build blunt-end libraries, following the method by[3]. Initial nebulization step was skipped, due to the high fragmentation of ancient DNA molecules. Each genomic library was amplified in six separate PCR reactions (each 25 µl) using primers and thermocycling conditions according to[4]. Six amplified samples were pooled and purified using AMPure® XP Reagents (Agencourt-Beckman Coulter) following the manufacturer's instructions. The fragment size distribution and concentration of each library was measured with High Sensitivity D1000 Screen Tape assay on 2200 TapeStation system (Agilent). Indexed DNA libraries were pooled in equimolar amounts and sequenced on Illumina HiSeq2500 (125bp, pair end, each library on 1/15 lane) at the Archaeological Research Laboratory (AFL), Stockholm, Sweden.

Ukrainian samples (*n*=8) processed in Stockholm underwent similar treatment, however less bone powder was used for DNA extraction (~80-110 mg) and purified libraries were quantified using DNA High Sensitivity Kit with Agilent 2100 Bioanalyzer Instrument. The samples were screened on Illumina HiSeq2500 (125bp PE; each library on 1/8 or 1/11 lane). Individuals SCY009 and SCY010 were further sequenced one individual per Illumina HiSeq2500 lane.

All Illumina sequencing was performed at the National Genomics Infrastructure (NGI) in Stockholm. Sequence data was merged and mapped to human genome using approach by[4]. All primary pipeline computations were performed on resources provided by the *Swedish National Infrastructure for Computing (*SNIC) through Uppsala Multidisciplinary Center for Advanced Computational Science (UPPMAX).

Mitochondrial genome capture

Prior to the hybridization step, the DNA libraries (each ~100 ng) were concentrated to dryness with the use of Speedvak concentrator (Savant) and resuspended in 6.8 µl of ddH_2O.

Hybridization capture reactions were performed using commercially biotinylated probes for human mtDNA provided by MYcroarray® (Ann Arbor, MI, USA; www.mycroarray.com). The reactions conducted at 60°C for 24h in a final volume of 30 µl. Captured mitochondrial targets were recovered with Dynabeads® MyOne Streptavidin C1 magnetic beads (Invitrogen), followed by bead-bait binding and beads washing according to the manufacturer's protocol. Post capture amplification, after the first round of enrichment, was performed in five separate reactions per sample, each comprising of: 1 µl of PCR primer IS5 (10 µM), 1 µl of PCR primer IS6 (10 µM), 10 µl of 2x HiFi HotStart ReadyMix (KAPA), 2 µl of H_2O and 6 µl of bead-bind DNA library. The thermocycling conditions consisted of an initial denaturation at 98°C for 2 min, 12 cycles of 98°C for 20 s, 60°C for 30 s, 72°C for 30 s, and final extension at 72°C for 5 min. After the second round of enrichment, post capture amplification was carried out in five separate reactions per sample, each comprising of: 1 µl of PCR primer PISI (10 µM), 1 µl of PCR primer AIS4 (10 µM), 10 µl of 2x HiFi HotStart ReadyMix (KAPA), 2 µl of H_2O and 6 µl of bead-bind DNA library. All primer sequences used in this study are shown in Supplementary Information Text Table S8. The thermocycling conditions were similar to those presented above, except the primer annealing temperature which was conducted in 57°C. After post capture amplification, the five amplified samples per reaction were pooled and purified with the use of MinElute spin columns (Qiagen) following manufacturer's instructions.

DNA concentrations and fragment length distribution of enriched, and indexed libraries were determined on 2200 TapeStation system (Agilent) following manufacturer's protocol. Indexed and enriched libraries were pooled to an equimolar concentrations and adjusted to a final concentration of 20 pM. Enrichment with positive Ion Sphere Particles (ISPs) was performed using the Ion Torrent One Touch System II and the Ion One Touch 200 template kit v2 DL. Sequencing with the template ISPs was conducted with the use of Ion Torrent Personal Genome Machine (Ion PGM) system (Ion Torrent, Thermo Fisher Scientific Inc.) at Molecular Biology Techniques Laboratory, Faculty of Biology, AMU. Sequencing was performed on the Ion 318™ Chip Kit v2 using 520 flows and the Ion PGM Hi-Q sequencing kit v2.

References

1. Yang, D. Y., Eng, B., Waye, J. S., Dudar, J. C. & Saunders, S. R. Technical note: improved DNA extraction from ancient bones using silica-based spin columns. *Am. J. Phys. Anthropol.* **105,** 539–543 (1998).
2. Malmström, H. *et al.* More on contamination: the use of asymmetric molecular behavior to identify authentic ancient human DNA. *Mol. Biol. Evol.* **24,** 998–1004 (2007).
3. Meyer, M. & Kircher, M. Illumina sequencing library preparation for highly multiplexed target capture and sequencing. *Cold Spring Harb. Protoc.* **2010,** pdb.prot5448 (2010).
4. Günther, T. *et al.* Ancient genomes link early farmers from Atapuerca in Spain to modern-day Basques. *Proc. Natl. Acad. Sci. U. S. A.* **112,** 11917–11922 (2015).

Data analysis

Bioinformatic analysis

Read pairs were merged, requiring an overlap of at least 11bp and summing up base qualities. MergeReadsFastQ cc.py was used to remove adapters according to[1]. Merged reads were mapped as single-end reads against the revised Cambridge Reference Sequence (rCRS) with the use of BWA software package version 0.7.8[2] using the "aln" option with the non-default parameters -l 16500 -n 0.01 -o 2 -t 2.

MtDNA data from PGM Ion Torrent was processed using a pipeline adjusted specifically to the Ion Torrent reads. Sequences were demultiplexed by barcode with one mismatch threshold using the fastx_barcode_splitter.pl and fastx_trimmer scripts (http://hannonlab.cshl.edu/fastx_toolkit/). Long (−M 110), short (−m 35), and low-quality sequences (−q 20) were removed using Cutadapt v.1.8.1[3]. The same script was applied to trim adapters with the use of a maximum error rate of 0.33 (−e 0.3333), for a total of five passes (−n 5). The filtered sequence reads were analyzed with FastQC v 0.11.3[4], followed by mapping against the rCRS using TMAP v3.4.1[5] with options: −g 3 -M 3 -n 7 -v stage1 –stage-keep-all map1 –seed-length 12 –seed-max-diff 4 stage2 map2 –z-best 5 map3 –max-seed-hits 10.

FilterUniqueSAMCons.py was used to create consensus sequences based on PCR duplicate reads with identical start and end coordinates, for both PGM and Illumina sequence data as in[1]. Misincorporation patterns were determined with the use of mapDamage v2.0.5[6]. Final sequences were visualized using Biomatters IGV software v2.3.66[7]. ANGSD v0.910[8] was applied to build consensus sequence accepting only reads with mapping score of 30, a minimum base quality of 20, and a minimum coverage of 3.

Mitochondrial haplotypes were determined for each sample with the use of HAPLOFIND[9] and PhyloTree phylogenetic tree build 17[10]. The mutations reported as unexpected or missing were visually inspected in original binary alignment map (BAM) files in IGV.

Ratio of reads mapping to Y and X chromosomes (R_y) was performed to determine molecular sex on Illumina sequences as showed by[11]. Individuals with R_y ratio ≤ 0.016 were identified as females, while males were determined when $R_Y \geq 0.077$ (Supplementary Information Text Table S7). The molecular sex calculation was restricted only to the DNA sequence reads with mapping qualities of at least 30.

Population genetic analyses

Comparative ancient samples used in the statistical analyses were retrieved form the web depositories (NCBI Nucleotide, European Nucleotide Archive) and literature, in a form of consensus FASTA files or bam files. In case of BAM files, consensus mtDNA sequences were reconstructed utilizing the SAMtools software[2]. Detail information about ancient populations used for comparative studies and their references is shown in Supplementary Tables S2-S4.
To calculate principal component analysis (PCA) we gathered mtDNA haplogroups (hg) frequencies from particular comparative ancient populations (Supplementary Table S2). Additionally, we included Asian origin mtDNA haplogroups: A, B, D, F, G and M hgs and their frequencies to cover suggested Asian influences into Scythian populations. PCA was computed using RapidMiner Studio 7 (RapidMiner Inc., Boston, MA, USA). The PCA results and variables of mtDNA hgs loadings were plotted using Matplotlib 1.5.1 Python package.

We have performed top-down clustering analysis in the form of k-means algorithm to explore the unbiased population relations in our "PCA based on HG frequencies" sample-set. We tested several distance based measures for the k-means procedure and chosen the one with highest between cluster distances and lowest within cluster distances – as measured by Davies Bouldin index[12]. Testing k from 3 to 9, there was a significant reduction in Davies Bouldin index at k=5 (using Mixed Euclidean distance). K-means analysis was performed in RapidMiner Studio 7 (RapidMiner Inc., Boston, MA, USA).

Pairwise genetic distances (F_{ST}) were applied only to samples with complete mitochondrial genome sequences. The list of comparative populations with whole mitochondrial genomes and their references is shown in Supplementary Table S3. F_{ST} was computed in Arlequin 3.5 software[13]. To reduce the chance of incorporating erroneous SNPs, the first and last 30 nucleotides from the mtDNA sequences were removed. Nei's average number of pairwise differences[14] between the populations was calculated with 1000 permutations and p-value of 0.05. Multidimensional scaling (MDS) was computed on the pairwise genetic distances between populations with the use of Python scikit-learn 0.17 package[15]. The maps were created using QGIS 2.12.2[16].

The median network was calculated for hg U5 using Networks 4.614 software (fluxus-engineering.com). The procedure included weighting the most common mutations inversely according to their frequency. For computations we used Reduced Median algorithm[17], followed by Median Joining algorithm[18]and maximum parsimony calculation (postprocessing) to reduce the superfluous links[19].

Pairwise distances were visualized using heatmap with geographic distributions and gradient colors representing the corresponding F_{ST} values (Fig. 4)**.** AMOVA analysis was run with the same population set that was used to compute F_{ST} pairwise genetic distances (whole mtDNA genomes, 12 populations). We have tested all combinations of grouping Scythians pairwise with other 11 populations.

References:

1. Meyer, M. & Kircher, M. Illumina sequencing library preparation for highly multiplexed target capture and sequencing. *Cold Spring Harb. Protoc.* **2010,** pdb.prot5448 (2010).
2. Li, H. & Durbin, R. Fast and accurate short read alignment with Burrows-Wheeler transform. *Bioinforma. Oxf. Engl.* **25,** 1754–1760 (2009).
3. Martin, M. Cutadapt removes adapter sequences from high-throughput sequencing reads. *EMBnet.journal* **17,** 10–12 (2011).
4. Andrews, S. . A quality control tool for high throughput sequence data. (2012).
5. Merriman, B., Ion Torrent R&D Team & Rothberg, J. M. Progress in ion torrent semiconductor chip based sequencing. *Electrophoresis* **33,** 3397–3417 (2012).
6. Jónsson, H., Ginolhac, A., Schubert, M., Johnson, P. L. F. & Orlando, L. mapDamage2.0: fast approximate Bayesian estimates of ancient DNA damage parameters. *Bioinforma. Oxf. Engl.* **29,** 1682–1684 (2013).
7. Robinson, J. T. *et al.* Integrative genomics viewer. *Nat. Biotechnol.* **29,** 24–26 (2011).
8. Korneliussen, T. S., Albrechtsen, A. & Nielsen, R. ANGSD: Analysis of Next Generation Sequencing Data. *BMC Bioinformatics* **15,** 356 (2014).
9. Vianello, D. *et al.* HAPLOFIND: a new method for high-throughput mtDNA haplogroup assignment. *Hum. Mutat.* **34,** 1189–1194 (2013).
10. van Oven, M. & Kayser, M. Updated comprehensive phylogenetic tree of global human mitochondrial DNA variation. *Hum. Mutat.* **30,** E386-394 (2009).

11. Skoglund, P., Stora, J., Götherström, A. & Jakobsson, M. Accurate sex identification of ancient human remains using DNA shotgun sequencing. *J. Archaeol. Sci.* **40,** 4477–4482 (2013).
12. Davies, D. L. & Bouldin, D. W. A cluster separation measure. *IEEE Trans. Pattern Anal. Mach. Intell.* **1,** 224–227 (1979).
13. Excoffier, L. & Lischer, H. E. L. Arlequin suite ver 3.5: a new series of programs to perform population genetics analyses under Linux and Windows. *Mol. Ecol. Resour.* **10,** 564–567 (2010).
14. Nei, M. & Li, W. H. Mathematical model for studying genetic variation in terms of restriction endonucleases. *Proc. Natl. Acad. Sci. U. S. A.* **76,** 5269–5273 (1979).
15. Pedregosa, F. *et al.* Scikit-learn: Machine learning in Python. *J Mach Learn Res* 2825–2830
16. QGIS Development Team. *QGIS Geographic Information System. Open Source Geospatial Foundation Project* http://www.qgis.org (2015).
17. Bandelt, H. J., Forster, P., Sykes, B. C. & Richards, M. B. Mitochondrial portraits of human populations using median networks. *Genetics* **141,** 743–753 (1995).
18. Bandelt, H. J., Forster, P. & Röhl, A. Median-joining networks for inferring intraspecific phylogenies. *Mol. Biol. Evol.* **16,** 37–48 (1999).
19. Polzin, T. & Daneshmand, S. V. On Steiner trees and minimum spanning trees in hypergraphs. *Oper Res. Lett.* **31,** 12–20 (2003).

DNA extraction and low-resolution PCR-RFLP analysis of mtDNA of Scythian specimens from Ukraine – Grand Valley State University (GVSU)

Specimens SCY6, SCY9, SCY10, and SCY11 were earlier analyzed at Grand Valley State University (GVSU) by low-resolution screening of the diagnostic coding and hypervariable 1 (HVR-1) regions of mtDNA followed by Sanger DNA sequencing. Two independent temporary separated extractions were performed for each specimen.

To ensure the authenticity of the results we adhered to the best practice procedures for working with aDNA (summarized in[1]) as much as possible. All aDNA manipulations were conducted in a dedicated limited-access aDNA facility with two laminate flow hoods equipped with HEPA air filtration and internal UVC light systems (one hood for DNA extraction, one for PCR setup). Full body coveralls, facemasks and face shields were worn at all times during all specimen manipulations. To further reduce the chances of contamination by modern DNA through handling, all pre-PCR specimen manipulations were performed by a single person.

Bone specimens were cleaned in a separate facility under a chemical flow hood by sanding off ~1mm of the surface to remove surface contamination. Specimens were irradiated on all sides using 253.5 nm UV light. About 1g of bone was removed per extraction using a Dremel tool and powdered using a sterilized porcelain mortar and pestle. The powder was washed three times with EDTA pH 8.0 followed by three rinses with sterile water, pH 7. DNA was extracted using a QIAGEN QIAmp DNA Investigator Kit (QIAGEN Inc., Valencia, CA, USA) following a modified QIAamp protocol for extraction of DNA from bone. Negative controls were used with each extraction. Extracted DNA was eluted in 20-25 µl of sterile water and stored at -20°C. Four overlapping primer pairs were used to amplify the HVR-1 and diagnostic coding regions using previously reported primers[2]. An additional primer pair (L16185: AACCCAATCCACATCAAAACC; H16273: AGGGTGGGTAGGTTTGTTGGTATCC) producing a 133-bp fragment was used to improve the resolution at nucleotide position 16189 of HVR-1. MtDNA diagnostic coding region sites for haplogroups H (nucleotide position 7028), J/T (nucleotide position 4216) and U (nucleotide position 12308) were amplified using previously published primers[3]. Negative controls were used to detect the presence of contamination and positive controls, set up in isolation from aDNA, were used to establish

effective PCR chemistry. Amplification was carried out using a QIAGEN Fast-Cycling PCR Kit as directed in the kit protocol following the conditions optimized for fragments in the 10-100-copy range. Amplification cycles were kept at 49 rounds. Each coding and control region segment was amplified up to four times per extraction or until two independent amplification products were obtained. Successful amplifications were cleaned using a Qiagen MinElute kit and eluted into 10µl of sterile water.

Successful amplifications were cloned by ligation into QIAGEN pDrive vectors using a QIAGEN PCR Cloning Kit. Transformed cells were grown on sterile LB-Amp agar plates and incubated at 37°C for 16 to 20 hours. Cells containing the PCR insert were selected by blue-white differentiation, re-plated and incubated again at 37°C for 20-26 hours. Subcultured cells were eluted into 250µL of sterile water using a sterile loop. Clone DNA amplification was performed by using 1µL of resuspended cells with SP6 and T7 universal primers to amplify the entire fragment within the plasmid cloning site. After an initial 5 minutes at 95°C to lyse cells, 29 PCR cycles were as follows: 94°C for 30 seconds, 42°C for 45 seconds, 72°C for 90 seconds with one elongation step of 72°C for 5 minutes at the end of the 29 cycles. To verify an insertion of the desired PCR fragment into the plasmid vector, PCR products were visualized on a 2.5% agarose gel.

Sanger DNA sequencing analysis was performed at the Annis Water Research Institute at GVSU. Sequencing reactions were carried out on 96-well plates using BigDye Terminator v3.1 Cycle Sequencing Kit (Applied Biosystems) for 45 rounds. Sequencing reactions were cleaned before sequencing using a standard Sephadex protocol. Samples were run on an ABI 3130x1 Genetic Analyzer with a 50-cm capillary array.

DNA sequence analysis was accomplished using the tools from NCBI BLAST (http://blast.ncbi.nlm.nih.gov/Blast.cgi) through alignment with the revised Cambridge Reference Sequence (rCRS) of mtDNA (GenBank accession # NC 012920) to determine SNP differentiation. SNP variations were referenced with the phylogenetic tree of global human mtDNA variation (phylotree.org), based on both coding and control region polymorphisms, to determine haplogroup assignment. All chromatograms were thoroughly inspected using the 4Peaks DNA sequence viewer program (A. Griekspoor and Tom Groothuis, mekentosj.com) and ambiguous base assignments were manually called by researcher.

To insure the authenticity of aDNA results, multiple criteria were utilized, including multiple extractions, DNA fragment length quantification based on Bioanalyzer data (aDNA is expected to be highly fragmented), fragment size-dependent amplification success frequency evaluation (shorter fragments should be preferentially amplified in an aDNA/ contamination mix), multiple PCR amplifications of the same region for each specimen, short length of amplified fragments (128 - 266 bp), overlapping fragment amplification, SNP match in overlapping fragments, and the cloning of amplified DNA fragments. The molecular behaviour of amplified fragments was examined. The sequences considered to be genuine aDNA displayed signs of deamination damage as well as having the potential for increased chimerization. Furthermore, SNP patterns from genuine aDNA were expected to make phylogenetic sense. An additional robust authenticity criterion was achieved by duplicate mtDNA analyses of the specimens at AMU and SU.

References:

1. Knapp, M., Clarke, A. C., Horsburgh, K. A. & Matisoo-Smith, E. A. Setting the stage - building and working in an ancient DNA laboratory. *Ann. Anat. Anat. Anz. Off. Organ Anat. Ges.* **194,** 3–6 (2012).
2. Nikitin, A. G., Newton, J. R. & Potekhina, I. D. Mitochondrial haplogroup C in ancient mitochondrial DNA from Ukraine extends the presence of East Eurasian genetic lineages in Neolithic Central and Eastern Europe. *J. Hum. Genet.* **57,** 610–612 (2012).
3. Santos, C. *et al.* Determination of human caucasian mitochondrial DNA haplogroups by means of a hierarchical approach. *Hum. Biol.* **76,** 431–453 (2004).

Supplementary Table S7. Molecular sex identification of the investigated individuals, where n_X is the number of sequences aligning to chromosome X; n_Y is the number of sequences aligning to chromosome Y, and R_Y ratio is calculated as $n_Y/(n_X + n_Y)$.

Sample	$(n_X + n_Y)$	n_Y	R_Y	*SE*	95% CI	Mol. Sex assignment
SCY001	533	48	0.0901	0.0124	0.0658-0.1144	consistent with XY but not XX
SCY002	1405	123	0.0875	0.0075	0.0728-0.1023	consistent with XY but not XX
SCY005	1321	6	0.0045	0.0019	0.0009-0.0082	XX
SCY006	3614	36	0.01	0.0017	0.0067-0.0132	XX
SCY009	630557	59405	0.0942	0.0004	0.0935-0.0949	XY
SCY010	177360	1178	0.0066	0.0002	0.0063-0.007	XX
SCY011	8347	48	0.0058	0.0008	0.0041-0.0074	XX
SCY012	809	71	0.0878	0.0099	0.0683-0.1073	consistent with XY but not XX
SCY192	54946	364	0.0066	0.0003	0.0059-0.0073	XX
SCY193	11866	1149	0.0968	0.0027	0.0915-0.1022	XY
SCY196	3248	249	0.0767	0.0047	0.0675-0.0858	consistent with XY but not XX
SCY197	43947	4278	0.0973	0.0014	0.0946-0.1001	XY
SCY300	29823	176	0.0059	0.0004	0.005-0.0068	XX
SCY303	96388	682	0.0071	0.0003	0.0065-0.0076	XX
SCY305	7840	697	0.0889	0.0032	0.0826-0.0952	XY
SCY308	3065	281	0.0917	0.0052	0.0815-0.1019	XY
SCY311	79377	632	0.008	0.0003	0.0073-0.0086	XX
SCY332	23185	184	0.0079	0.0006	0.0068-0.0091	XX
SCY334	396	31	0.0783	0.0135	0.0518-0.1047	consistent with XY but not XX

Supplementary Table S8. Description of primer sequences used in the studies.

Name	Primer sequences (5’→3’)	Reference
IS4	AATGATACGGCGACCACCGAGATCTACACTCTTTCC CTACACGACGCTCTT	[1]
IS5	AATGATACGGCGACCACCGA	[1]
IS6	CAAGCAGAAGACGGCATACGA	[1]
Indexing* primer	CAAGCAGAAGACGGCATACGAGATxxxxxxxGTGACT GGAGTTCAGACGTGT	[1]
PISI	CCTCTCTATGGGCAGTCGGTGACCTACACGACGCTCT TCCGATCT	This study
AIS4	CCATCTCATCCCTGCGTGTCTCCGACTCAGCAAGCAG AAGACGGCATACGAGAT	This study

*“xxxxxxx” is 7bp index, one of 228 different indexes included in Mayer and Kircher (2010)

1. Meyer, M. & Kircher, M. Illumina sequencing library preparation for highly multiplexed target capture and sequencing. *Cold Spring Harb. Protoc.* **2010,** pdb.prot5448 (2010).

SCY012

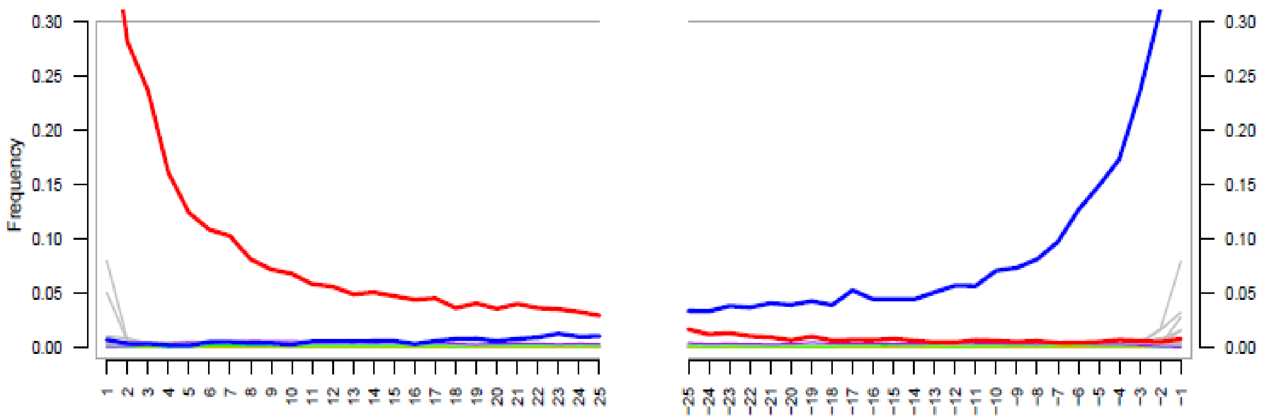

SCY011

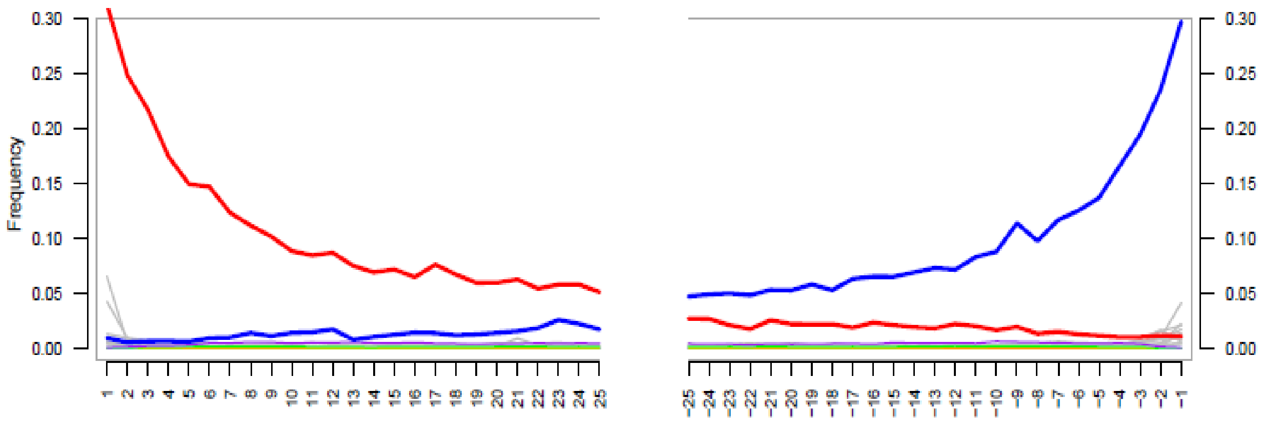

SCY006

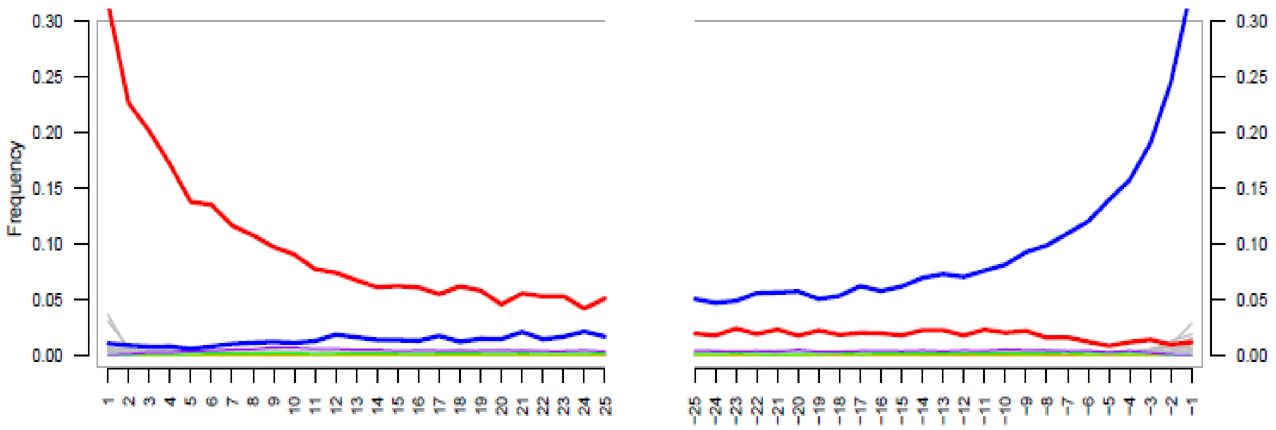

SCY002

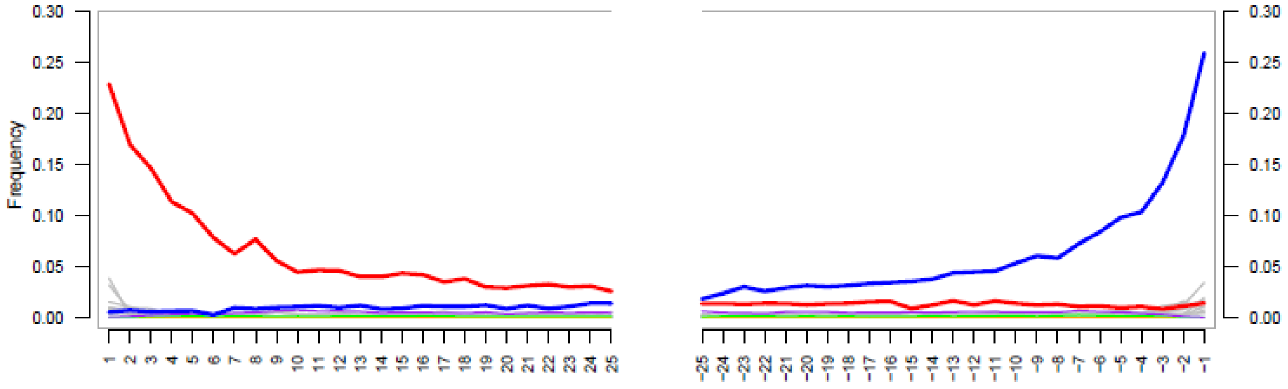

SCY001

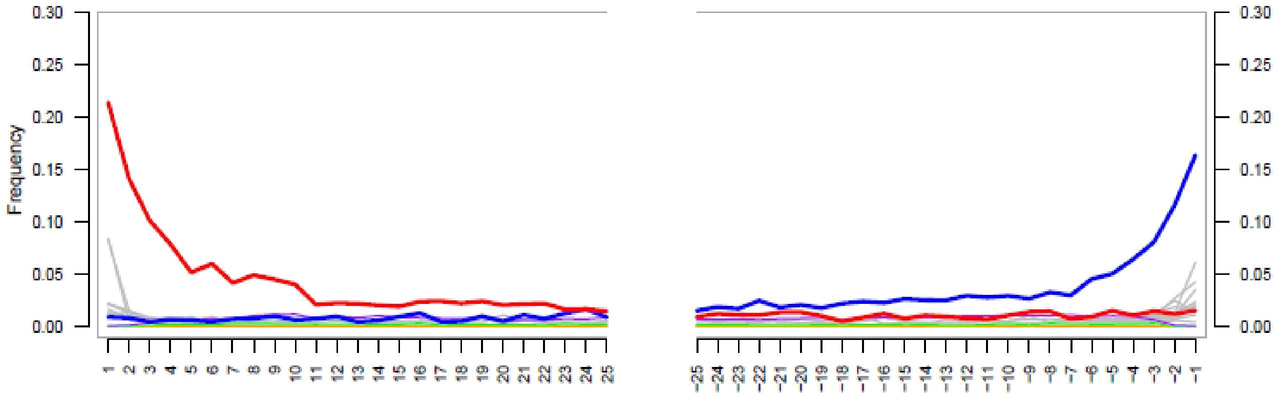

SCY 303

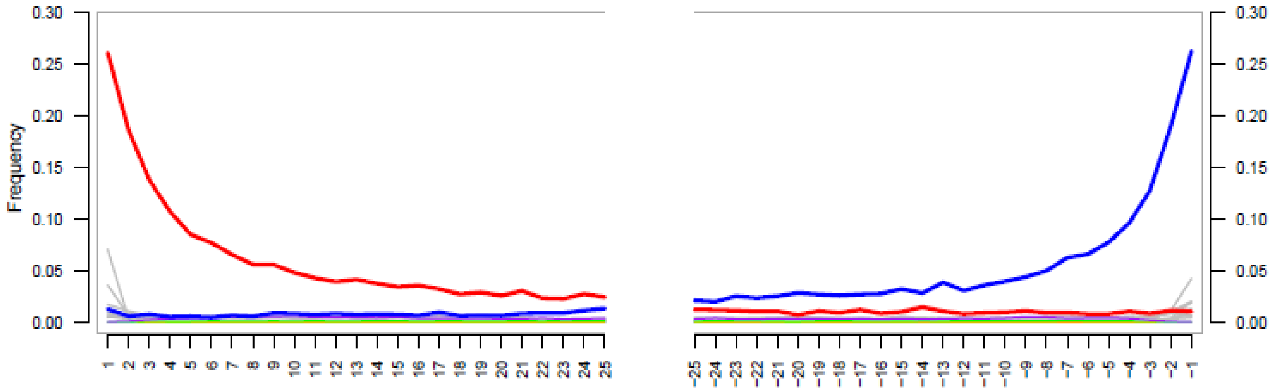

SCY192

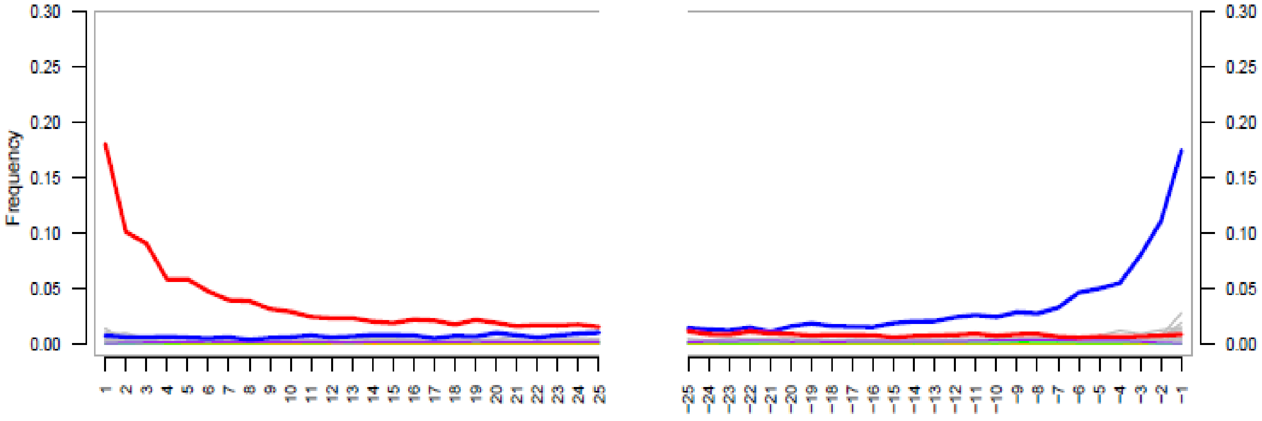

SCY197

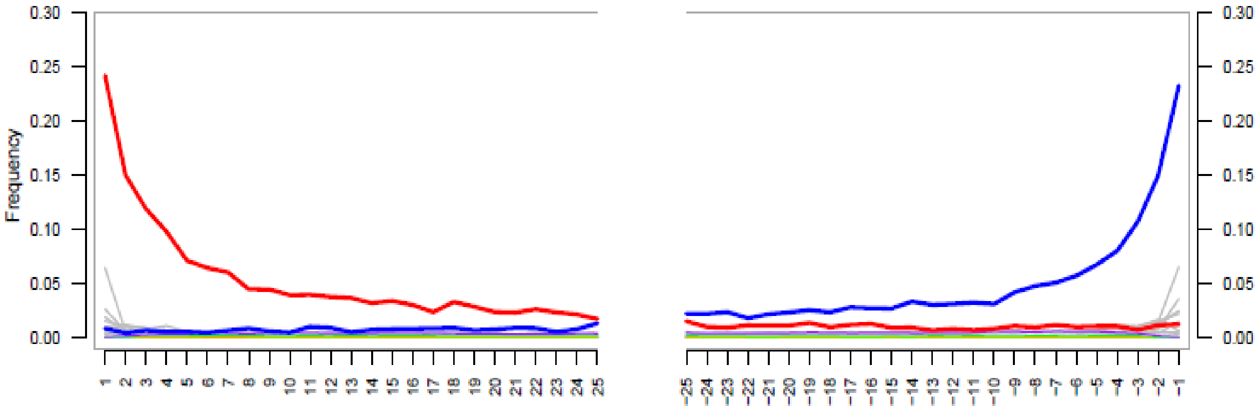

SCY193

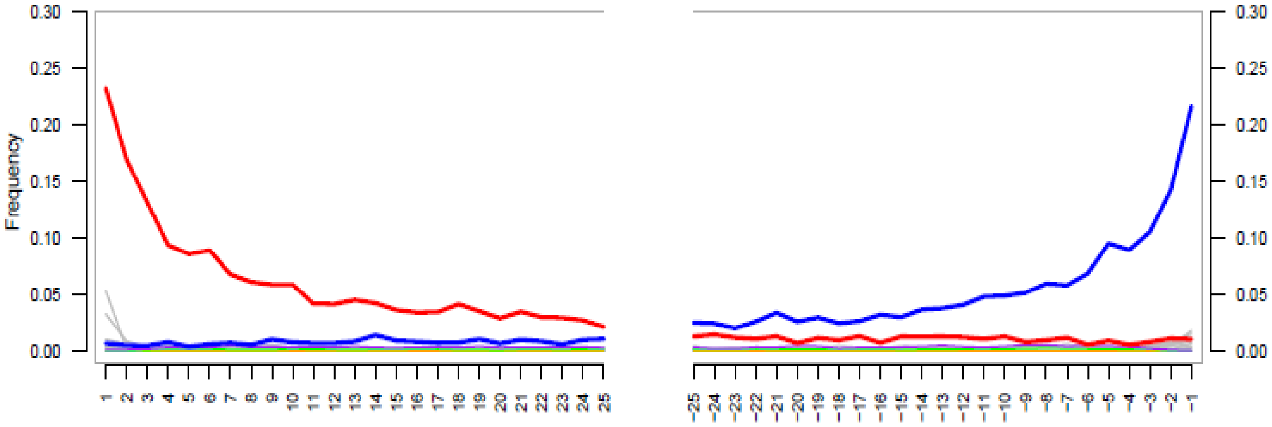

SCY300

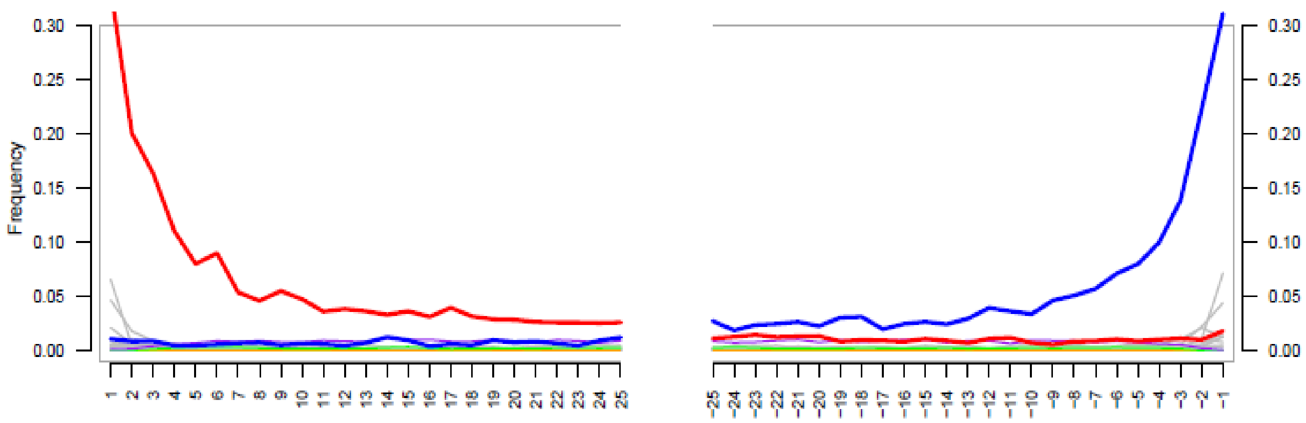

SCY305

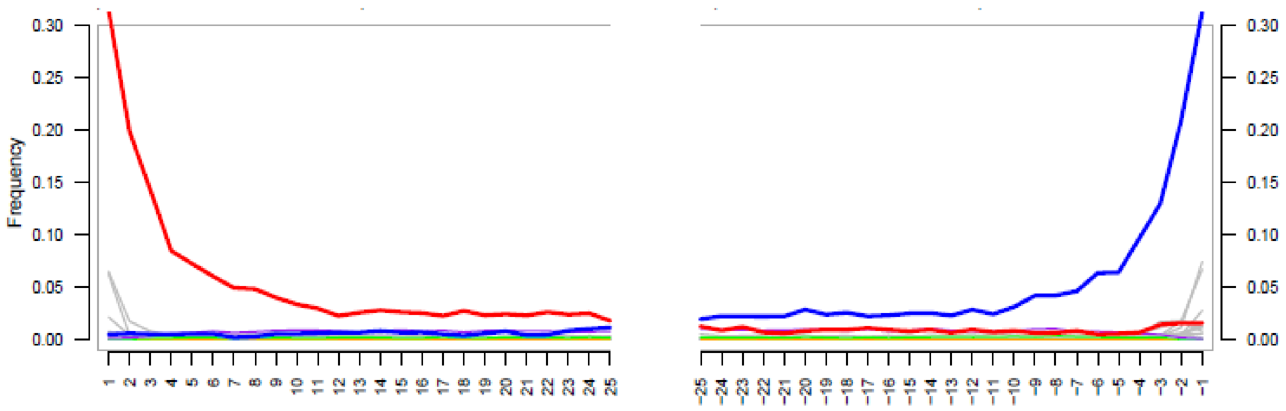

SCY308

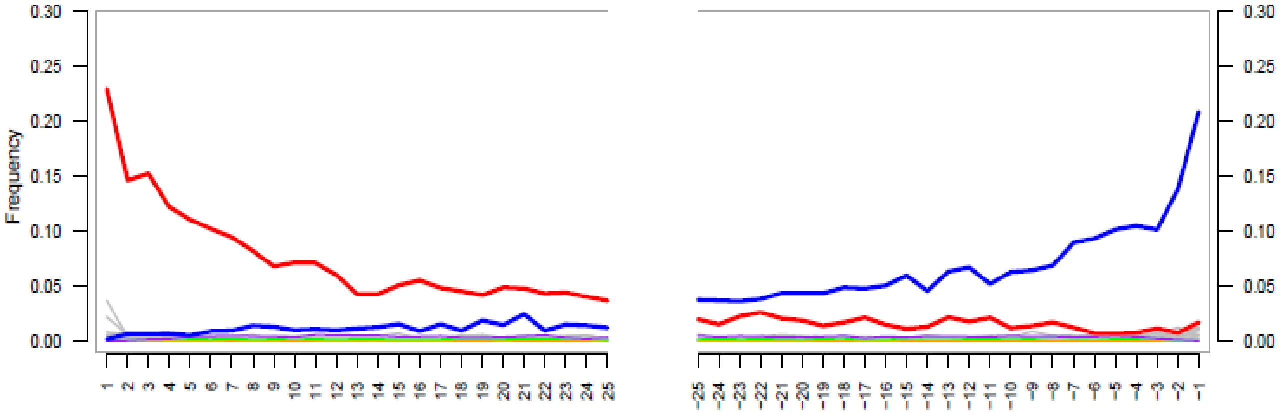

SCY311

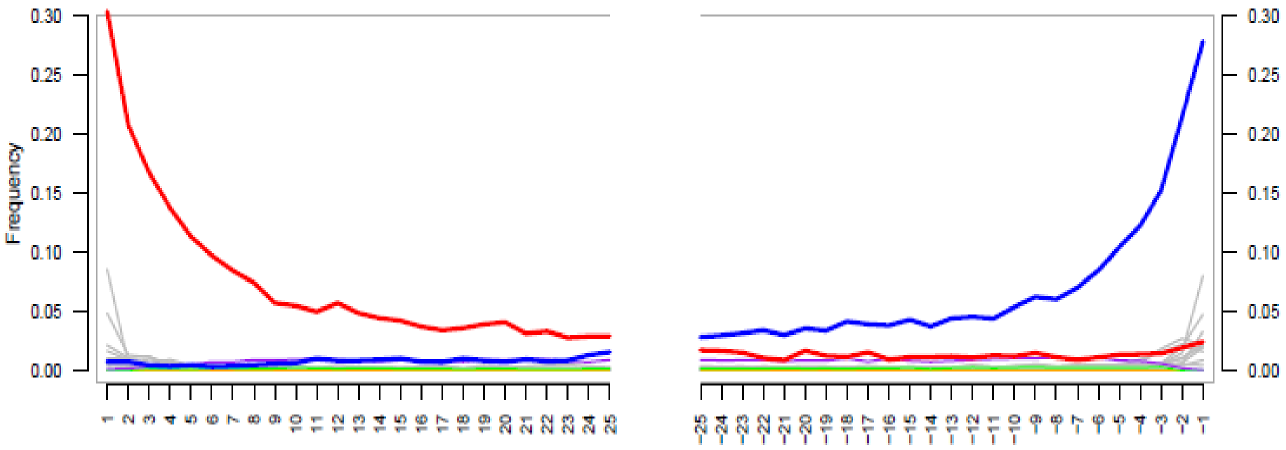

SCY332

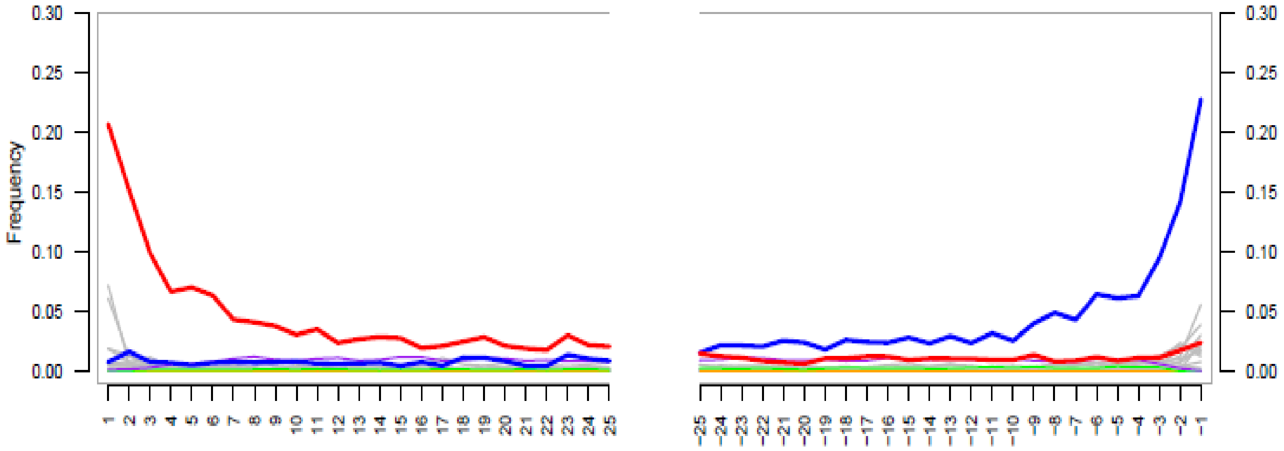

SCY334

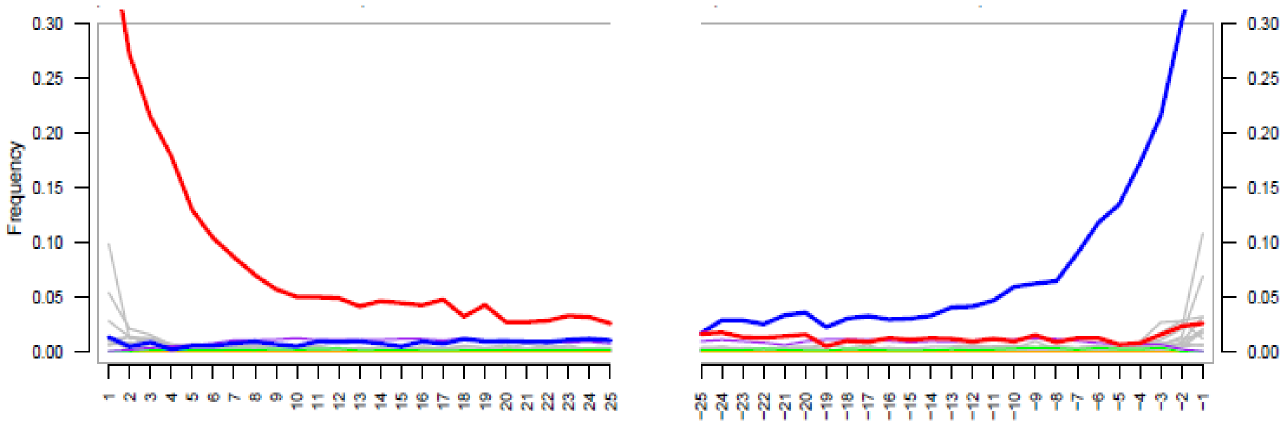

SCY196

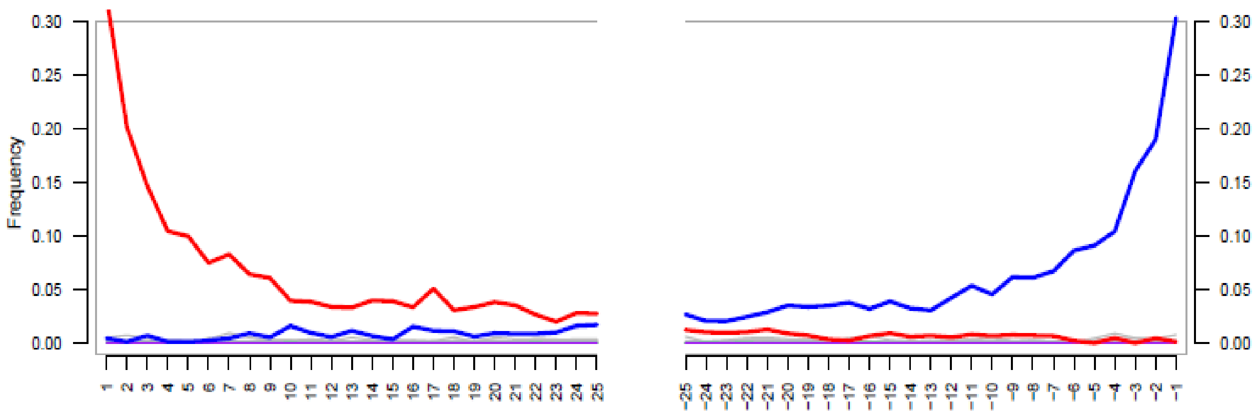

Supplementary Fig S11. mapDamage fragment misincorporation plots of Scythians: frequency of C to T transitions (red) at the 5'ends of reads (left) and frequency of G to A transitions (blue) at the 3'ends of reads (right).
